# Nanoparticles in the diagnosis and treatment of vascular aging and related diseases

**DOI:** 10.1038/s41392-022-01082-z

**Published:** 2022-07-11

**Authors:** Hui Xu, Shuang Li, You-Shuo Liu

**Affiliations:** 1 Department of Geriatrics, The Second Xiangya Hospital of Central South University, 410011 Changsha, Hunan China; 2 Institute of Aging and Age-related Disease Research, Central South University, 410011 Changsha, Hunan China

**Keywords:** Drug delivery, Cardiology, Cardiovascular diseases, Senescence, Gene therapy

## Abstract

Aging-induced alternations of vasculature structures, phenotypes, and functions are key in the occurrence and development of vascular aging-related diseases. Multiple molecular and cellular events, such as oxidative stress, mitochondrial dysfunction, vascular inflammation, cellular senescence, and epigenetic alterations are highly associated with vascular aging physiopathology. Advances in nanoparticles and nanotechnology, which can realize sensitive diagnostic modalities, efficient medical treatment, and better prognosis as well as less adverse effects on non-target tissues, provide an amazing window in the field of vascular aging and related diseases. Throughout this review, we presented current knowledge on classification of nanoparticles and the relationship between vascular aging and related diseases. Importantly, we comprehensively summarized the potential of nanoparticles-based diagnostic and therapeutic techniques in vascular aging and related diseases, including cardiovascular diseases, cerebrovascular diseases, as well as chronic kidney diseases, and discussed the advantages and limitations of their clinical applications.

## Introduction

Age is the most important risk factor for vascular aging and related disorders.^1^ Aging-induced alterations of vasculature functions, structure, and phenotypes play a pivotal role in the initiation and progression of various vascular aging-related diseases, such as cardiovascular, cerebrovascular, and kidney diseases.^2^ Age-related pathological alterations of the vasculature are tightly associated with vascular disorders.^3^ Multiple molecular and cellular events, such as inflammation, cell proliferation, migration, angiogenesis, thrombosis, and apoptosis contribute to vascular cell senescence.^4^ Vascular aging is predominantly characterized by endothelial cells (ECs) senescence and vascular smooth muscle cells (VSMCs) senescence. In line with the United Nations (2017) report on World Population Prospects, approximately 962 million people are aged 60 years and above, accounting for 13 percent of the global population.^5^ Currently, cardiovascular and cerebrovascular diseases are the leading causes of disability and mortality among older adults.^6^ Globally, vascular aging-related diseases have led to a significant social and economic burden.^7^ However, a lack of efficient diagnostic and curative strategies is a major challenge in the clinical management of vascular aging and related diseases. The diagnosis of vascular diseases is primarily determined by detecting biomarker levels and angiography, which are costly with low sensitivity.^8^ Several therapeutic options, such as genes, antisense drugs, peptides, and proteins, have been produced to treat vascular aging-related disease, however, many of them have limited efficacies or adverse side effects, which are attributed to poor stability, low bioavailability, rapid enzyme degradation, and off-targets.^9^ Healthy lifestyle behaviors, including regular exercise and dietary patterns, are effective strategies for preventing vascular aging. However, the majority of older adults do not meet the required healthy exercise or recommended diet thresholds.^10^ Therefore, the development of effective and reliable diagnostic and therapeutic modalities for vascular aging and related diseases is of utmost significance.

**Fig. 1:**
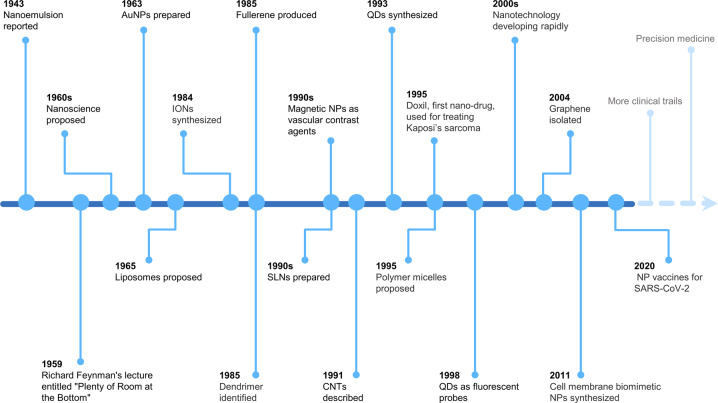
Timeline of the discovery and research history of nanoparticles. Key discoveries are highlighted. Research on nanoparticles began in the 1960s. Over the last two decades, an increasing number of scientists have devoted themselves to the study of nanoparticles, yielding impressive results in the biomedical field. AuNPs gold nanoparticles, IONs iron oxide nanoparticles, NPs nanoparticles, SLNs solid lipid nanoparticles, CNTs carbon nanotubes, and QDs quantum dots

Nanoparticles are microscopic particles that measure 1–100 nm in size, with various applications in the biomedical field.^11^ Nanoparticles integrating diagnostic and therapeutic agents into nanoparticle formulations have exerted comprehensive applications in various disorders, such as cancers,^12^ neurological diseases,^13^ cardiovascular diseases,^14^ liver diseases,^15^ and even kidney diseases.^16^ Diagnostic and therapeutic nanoparticles have been used to enhance the diagnostic as well as therapeutic efficacies and to reduce the incidences and intensities of side effects by increasing drug accumulations at pathological sites while decreasing drug accumulation in healthy tissues.^17,18^ Elucidation of the advances in nanoparticle research will inform the multifaceted clinical effects of nanoparticles (Fig. 1). Research on nanoemulsion has a long scientific history, beginning in 1943 when Hoar and Schulman first discovered and reported this dispersion system.^19^ Richard Feynman's 1959 lecture entitled "Plenty of Room at the Bottom" is a seminal event in the history of nanoscience and nanotechnology.^20^ In 1963, Uyeda et al. prepared gold nanoparticles (AuNPs) by evaporation in argon gas at low pressure.^21^ Liposomes, which were first proposed by Bangham et al. in 1965, have unique permeability and retention effects that make them novel drug delivery systems. Dendrimers were first identified and successfully synthesized by Tomalia in 1985.^22^ In the middle 1980s, Gleiter et al. successfully synthesized iron nanoparticles through inert gas condensation, marking a new era of research in nanoscience and technology.^23^ Magnetic nanoparticles were developed as vascular contrast agents for molecule imaging in 1990s.^24^ Carbon-based nanoparticles such as fullerene and carbon nanotubes (CNTs) were developed in 1985 and 1991, respectively.^25,26^ In 1993, Murray et al. synthesized homogenous quantum dots (QDs) in an organic solution.^27^ In tandem with advances in nanoscience, as fluorescent probes in biological staining and diagnostics, QDs were first reported in 1998.^28^ Doxil, doxorubicin encapsulated in lipid-based nanoparticles, was the first nanoparticle formulation to be approved by the Food and Drug Administration (FDA) in 1995 to treat Kaposi’s sarcoma.^12^ Solid lipid nanoparticles (SLNs) and polymeric micelles, which were first developed in 1990s, have been proposed as new generations of drug delivery systems.^29,30^ Since 2000, studies have investigated the potential applications of nanoparticles in diagnostics, imaging, gene, and drug delivery. Certain drugs such as dalargin, loperamide, or tubocurarine loaded onto polymeric nanoparticles exhibited various effects on the central nervous system.^31^ Graphene has been theoretically investigated since the 1940s and its existence has been known since the 1960s, however, it was not until 2004 when Geim and Novoselov completed its isolation that it attracted great scientific interest and became one of the most studied materials.^32,33^ The origins of cell-membrane biomimetic nanoparticles date back to 2011, when Hu et al. first reported erythrocyte membrane-camouflaged polymeric nanoparticles as biomimetic delivery platforms for achieving long-term circulation and targeted delivery.^34^ The significance of nanoparticles in the diagnosis and treatment of diseases has been widely investigated, with promising outcomes in drug delivery and diagnostic imaging.^35,36^ Critical effects of nanoparticles in vascular physiology and pathology have been reported, which supports their promise as advanced strategies for the management of vascular aging-related diseases. However, a comprehensive review of the applications of nanoparticles in vascular aging and related diseases has not been reported. Therefore, the principal purpose of this review is to explore the potential of nanoparticles in the diagnosis and treatment of vascular aging and related diseases, including cardiovascular diseases, cerebrovascular diseases, and chronic kidney diseases. Moreover, we discuss the advantages, limitations, several technical issues, and future work of nanoparticles.

## Classification of nanoparticles

In the past couple of decades, advances in nanotechnology have witnessed massive developments. Nanoparticles, which are less than 100 nm in size, have excellent functions, such as reactivity, roughness, and high surface energy through their physical and optical privileges.^37^ Nanoparticles such as AuNPs,^38^ CNTs,^39^ liposomes,^40^ dendrimers,^41^ micelles,^42^ and poly lactic-co-glycolic acid (PLGA)^43^ are promising in the field of diagnosis and treatment of vascular diseases (Table 1). During the coronavirus disease 2019 (COVID-19) pandemic, functionalized nanoparticles were used as nanoprobes to test nucleic acids.^44^ Different types of nanoparticles are emerged as drug delivery vehicles and diagnosis tools in vascular aging and related disorders. In this section, we provide an overview of current knowledge on the classification of nanoparticles, including inorganic-based, carbon-based, lipid-based, polymeric, and biomimetic nanoparticles.

**Table 1:** Nanoparticles utilized for research of vascular aging-related diseases

Nanoparticles	Subclasses	Construction	First synthesis	Advantages	Drawbacks	Ref(s)
Inorganic-based nanoparticles	AuNP	Comprise 102 gold atoms and 44 p-mercaptobenzoic acids	1963	Optical, biocompatibility, plasmon characteristics, physicochemical stability, surface chemistry, and multi-functionalization	Toxicity issues	^558^
	ION	γ-Fe_2_O_3_ or Fe_3_O_4_ core and a protective coating	1980s	Superparamagnetic, tissue permeability, biocompatibility, colloidal stability, and eco-friendliness	Toxicity, complex preparation process, and cost of scale-up production	^559^
	MSN	Pore diameter ranging from 2 to 50 nm	1992	High uniform pore passage, large surface area, narrow pore diameter distribution, and wide range	Genotoxicity, potential drug degradation, and time-consuming	^66,560^
	QD	Nanoscale semiconductor crystals	1981	Excellent chemical and photo-stability, high quantum yield, and size-tunable light emission	Environmental impact, manufacturing costs, overall toxicity, body clearance	^49^
Carbon-based nanoparticles	CNT	Multiple coaxial tubes composed of hexagonal carbon atoms	1991	High surface areas, superior adsorption ability, unique fluorescence, and Raman spectroscopy in the near-infrared region	Poor solubility, low biodegradability, low dispersivity, and toxicity problems	^561^
	Fullerene	Soccer ball-shaped hollow sphere formed by pentagonal and hexagonal rings of carbon atoms	1985	Good water solubility, large specific surface area, high specialized nanostructures, and electron affinity	Biodistribution and toxicity	^562^
	Graphene	Two-dimensional monolayer of sp^2^ hybridized carbon atoms bonded covalently in a hexagonal lattice	2004	Exceptionally high mechanical strength, high light transmittance, excellent electrical conductivity, and remarkable optical property	Cell viability and toxicity problems	^563^
	CQD	Carbon-based zero-dimensional nanoparticles composed of dispersed spherical carbon particles	2004	Favorable water dispersion, strong chemical inertia, and stable optical performance	Concentration-dependent biocompatibility	^564^
Lipid-based nanoparticles	Liposome	Lipid-based spherical vesicles in which lipophilic bilayer is sandwiched between two hydrophilic layers	1965	Hydrophilic and lipophilic, superior solubility, increased half-life, selective delivery, biocompatibility, and biodegradability	Increased cost, rapid clearance, some technical issues in sterility and shelf life, and toxicological and inflammatory effects	^565^
	SLN	Tiny and spherical particle composed of solid lipids	1990s	High surface area, tiny size, biocompatibility, biodegradability, and physical stability	Low drug loading capacity and drug expulsion under storage conditions	^566^
	NLC	Made of solid and liquid lipids	1990s	Good drug entrapment efficiency, higher drug loading capacity, higher drug stability, lower drug expulsion during storage, and better solubility	Stability issues, polymorphism, and storage problems	^566^
	Nanoemulsion	Biphasic dispersion of two immiscible liquids	1943	High stability, good taste experience, better affinity, long shelf life, and improved bioavailability	Safety and toxicity	^567^
Polymeric nanoparticles	Polymersomes	Self-assembled polymer shells composed of block copolymer amphiphiles	1990s	Larger molecular weights and structures, higher stability, and greater cargo-retention efficiency	Manufacturability, low encapsulation efficiency	^115^
	Micelles	Self-assembled monolayer	1995	High structural stability, high water solubility, customized and tailored to specific needs and separated functionality	Poor drug incorporation in some cases, toxicity, and unfavorable immunological interactions	^118^
	Dendrimer	Nanometric molecules that are radially symmetric, globular, mono-dispersed and homogenous	1985	Increase solubility, promoted absorption, high bioavailability, high penetrability, and targeted distribution	Cytotoxicity, hematological and immunological toxicity, and neurological toxicity	^568^
Biomimetic nanoparticles	Cell-membrane coated nanoparticles	Various cell membranes, such as RBCs, platelets, immune cells, and EVs	2011	Prolong systemic circulation, targeting specificity, high biocompatibility, low side effects, and immune escape	Undesirable side effects, induce or aggravate inflammation	^569^
	Nanoparticles with targeting ligands	Ligands include antibodies, antibody fragments, peptides, and other small molecules	1980s	High binding affinity, specificity, good biocompatibility, high stability, and low immunogenicity	High cost, high cost, limited stability, and low penetrability	^570^
	Natural protein-based nanoparticles	Proteins such as albumin, gelatin, lipoprotein, and ferritin proteins	2006	Biocompatibility, biodegradability, stability, surface modification of particles, and ease of particle size control	Rapid degradation, high cost, low yield, and batch-to-batch variation	^571^

*AuNP* gold nanoparticles, *ION* iron oxide nanoparticle, *γ-Fe*_*2*_*O*_*3*_ Maghemite, *Fe*_*3*_*O*_*4*_ magnetite, *MSN* mesoporous silica nanoparticle, *QD* quantum dot, *CNT* carbon nanotube, *CQD* carbon quantum dot, *SLN* solid lipid nanoparticle, *NLC* nanostructured lipid carrier, *RBC* red blood cell, *EV* extracellular vesicle

### Inorganic-based nanoparticles

**Fig. 2:**
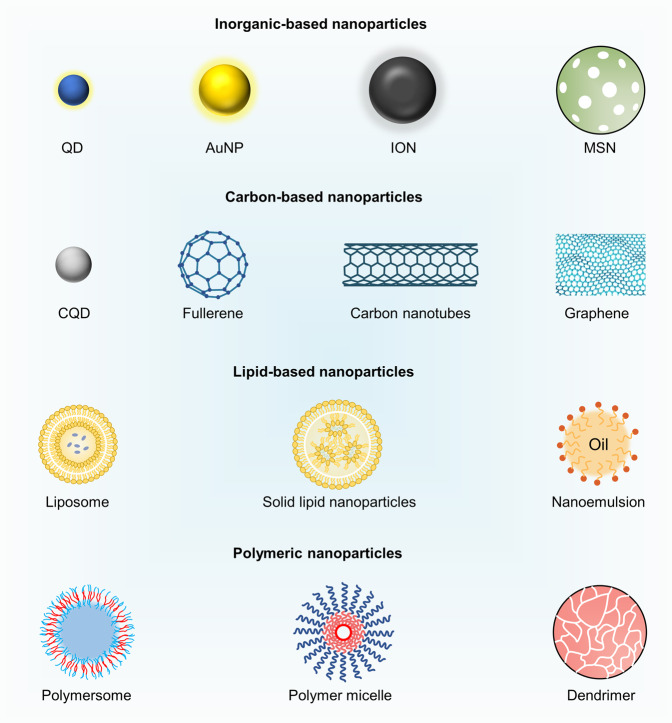
Schematic illustration of various inorganic-based nanoparticles, carbon-based nanoparticles, lipid-based nanoparticles, and polymeric nanoparticles. QD quantum dot, AuNP gold nanoparticle, ION iron oxide nanoparticle, MSN mesoporous silica nanoparticle, CQD carbon quantum dot

Given their unique physical, electrical, optical, and magnetic properties, inorganic-based nanoparticles have attracted considerable interest in biomedical applications.^45^ These inorganic nanoparticles are precisely formulated and can be designed in various sizes, structures, and geometry.^46^ Inorganic-based nanoparticles, such as AuNPs, iron oxide nanoparticles (IONs), mesoporous silica nanoparticles (MSNs), and QDs are ideal candidates for drug delivery and molecular imaging applications^47–49^ (Fig. 2).

AuNPs, which are among the well-studied nanoparticles, are synthesized in diverse sizes and shapes, such as spheres, cubes, rods, polygons, cages, prisms, bipyramids, and stars.^50^ AuNPs exhibit excellent properties, including biocompatibility, optical and plasmon characteristics, tunable physicochemical stability, low toxicity, controlled drug release, and easy functionalization and fabrication.^51^ Besides, as metallic nanoparticles, AuNPs have a variety of catalytic activities, such as esterase,^52^ nuclease,^53^ oxidase,^54^ peroxidase,^55^ superoxide dismutase,^56^ reductase,^57^ and catalase activities.^58^ Functionalized AuNPs are highly attractive and promising candidates in biological and biomedical applications, where they can be used as biosensors, in bioimaging, and as drug vehicles.^59^

IONs are a type of inorganic nanoparticles that have been extensively researched. Magnetic IONs, including maghemite (Fe_2_O_3_) or magnetite (Fe_3_O_4_) exhibit superparamagnetic properties with important applications in bioengineering and biomedical fields, where they can be used as contrast agents and drug carriers.^60^ The properties of IONs are highly correlated with their compositions, sizes, and shapes. Due to their unique magnetic properties, biocompatibility, stability, and eco-friendliness, IONs are excellent platforms for biomedical applications.^61^ In addition to acting as drug delivery systems, IONs are commonly fabricated to be bioimaging systems for use as contrast agents in magnetic resonance imaging (MRI) and magnetic particle imaging (MPI).^62^

MSNs are a group of nanoparticles with pore diameters of 2–50 nm.^63^ Their sizes, shapes, pore sizes, and pore volumes can be highly controlled. High surface areas and large pore volumes of MSNs provide ample biomolecule binding sites.^64^ In addition, their physicochemical and mechanical properties allow them to be promising carriers of various cargo, such as proteins and nucleic acids.^65^ Importantly, MSNs have abundant silanol groups on their surfaces, which can be modified to achieve controlled drug delivery, absorption, and release.^66^ Moreover, MSNs can be developed as biosensors and used for optical imaging and MRI.^67^

QDs, which are fluorescent semiconductor nanoparticles, are made up of hundreds to a few thousand atoms.^49^ Cores of QDs are only 2–10 nm in sizes, which can be replaced with AuNP or ION to mitigate long-term toxicities of QDs. Besides, QDs can be incorporated within larger carriers, such as liposomes and polymeric nanoparticles to serve as tracers.^68^ QDs released from larger carriers can mimic redistribution and eventual clearance of free drugs.^69^ Thus, QDs are a versatile platform for the design and development of nanoparticle-based drug vehicles.^70^

### Carbon-based nanoparticles

Carbon-based nanoparticles, such as CNTs, fullerene, graphene, and carbon quantum dots (CQDs) have been widely explored for various applications including bioimaging, biosensing, and drug delivery. These applications are attributed to their mechanical, electrical, thermal, and physicochemical properties as well as biological abilities.^71–73^

CNTs consist of carbon atoms arranged in condensed benzene rings. Based on their unique mechanical, electronic, optical, high elastic moduli, light weight, and stability properties, CNTs are of great clinical significance.^74^ CNTs can be grouped into single-walled CNTs (SWCNTs) and multi-walled CNTs (MWCNTs). SWCNTs, which consist of single graphene cylinders, are seamless cylindrical tubes with diameters of 0.4–2 nm, while MWCNTs are concentric tubes comprising multiple graphene sheet layers with inner diameters of 1–3 nm and outer diameters of about 2–100 nm.^75,76^ CNTs, with diameters of about 1 nm and lengths of several micrometers, have high aspect ratios and large surface areas.^77^ Thus, they provide multiple binding sites and improved cellular uptake. Hollow interiors of CNTs can be loaded with drugs and can maintain sustained drug release while avoiding degradation.^75^

Fullerene, also known as Buckyball or Buckminsterfullerene, is a closed hollow cage carbon molecule consisting of pentagonal and hexagonal rings of carbon atoms in which carbon atoms are sp^2^ hybridized.^78^ Fullerene, with specific geometry, sizes, and surfaces, exhibit unique spherical structures and physicochemical properties.^79^ Investigation of the significance of fullerene in biomedical applications is inhibited by its insolubility in water and organic solvents. Functionalization of fullerene is a constructive strategy to promote its water solubility and hydrophilicity.^80^ Fullerenes have been described as “radical sponges”.^81^ For instance, poly(l-glutamic acid) (PLE)-attached fullerenes can dose-dependently scavenge for free radicals.^82^

Graphene, the thinnest and strongest material, is a carbon-based two dimensional atomic crystal comprising a single-layer array of sp^2^ hybridized carbon arranged in a honeycomb lattice and exhibits satisfactory effects, stable quality, metallic, and high stiffness.^83^ The large surface area of graphene provides abundant binding sites for biomolecules while interactive functional groups (–COOH, –OH, and –COC) promotes its functionalization with other molecules.^84^ Given its thermal, mechanical, and electrical properties, large surface area, and versatile surface functionalization, graphene has gained substantial interest in drug delivery, bioimaging, and biosensing of vascular aging and related diseases.^85^

CQDs, with a carbon-based skeleton and many oxygen-containing groups, were accidentally discovered via a top-down technique in 2004.^86^ The average size of CQD is 3 nm.^78^ Due to their unique structures, CQDs can be dispersed in water and possess superior emission properties and chemical stability. Besides, CQDs have excellent biocompatibility and low cytotoxicity properties. CQDs have various biomedical applications, including biosensing, bioimaging, and biomedicine.^87^ Furthermore, due to their advanced optical characteristics, they are promising candidates for future optoelectronic applications, compared to other carbon-based nanoparticles.^88^

### Lipid-based nanoparticles

Lipid-based nanoparticles have been successfully used in the field of nanomedicine with a great deal of attention in vascular aging and related disorders.^89^ These nanoparticles, such as liposomes, SLNs, nanostructured lipid carriers (NLCs), and nanoemulsion have been recognized as outstanding drug carriers.^90,91^

Liposomes, which are spherical nanoparticles, are composed of phospholipids.^46^ Since their discovery in 1965, liposomes have developed tremendous investigations.^92^ Given the hydrophilic and lipophilic properties of phospholipids, liposomes can carry and deliver hydrophilic, hydrophobic, and lipophilic compounds.^93^ Besides, the ability of liposomes to encapsulate solutes and their selective release makes them attractive drug delivery systems.^94^ The stability of liposomes is highly associated with nanoparticle sizes, surface charge, and lipid composition.^46^

SLNs, with average sizes of 10 to 500 nm, are mainly composed of physiological lipids.^95^ They have large surface areas and tiny sizes, making them suitable candidates as drug carriers.^95^ SLNs can load both hydrophobic and hydrophilic drugs.^96^ They are characterized by good biocompatibility, biodegradability, physical stability, controlled drug release, protection of labile drugs, prolonged release of drug molecules, specific targeting, low toxicity, easy availability, and the possibility of large-scale manufacture.^97,98^ However, SLNs present some obstructions, including low drug loading capacities and drug expulsions in storage conditions.^99^

NLCs are second-generation lipid-based nanoparticle formulations that were first developed in 1999.^100^ They are composed of solid and liquid lipids, dispersed in aqueous phases containing surfactants.^101^ Their average sizes are between 10 and 1000 nm. Drugs can be encapsulated in lipid-based nanoparticles, such as SLNs and NLCs for multiple administration routes, including oral, intravenous, topical, transdermal, ocular, pulmonary, and parenteral.^96,102^ Compared to SLNs, NLCs possess higher drug entrapment efficiencies, higher drug loading capacities, higher drug stabilities, lower drug expulsion during storage, and better solubility,^103^ thus, they are promising drug carriers in vascular aging-related diseases.^104–106^

Nanoemulsion, also referred to as ultrafine emulsion, submicron emulsion, and miniemulsion, is a class of thermodynamically stable and transparent dispersions of oil and water.^107^ Nanoemulsions are heterogeneous systems composed of two immiscible liquids, in which one (dispersed phase) is dispersed in form of nanoscale droplets in the other liquid (continuous phase) and stabilized by an emulsifier or surfactant.^108^ Droplet sizes range between 20 and 500 nm.^109^ Notably, stability, appearance, and rheology of nanoemulsion are determined by size, composition, concentration, and surface properties of dispersed droplets.^108^ Besides, small particle sizes, large surface areas, and low surface tension of nanoemulsion allow its excellent reactivity to surroundings. Due to higher solubilization, long-term stability, longer shelf life, and ease of preparation, nanoemulsions are widely used as hydrophobic molecule carriers.^110^

### Polymeric nanoparticles

Polymeric nanoparticles are ideal drug delivery platforms with the ability to optimize therapeutic strategies of vascular aging-related disorders.^111^ Based on their different morphologies and compositions, polymeric nanoparticles are divided into nanocapsules and nanospheres.^46^ Nanocapsules are reservoir systems with vesicular structures surrounded by a polymeric membrane or shell while nanospheres are solid matrix systems.^112^ Given the presence of oil core in nanocapsules, drugs are commonly dissolved. In contrast, the absence of oil in nanospheres leads to a continuous polymeric network in which the drugs can be entrapped inside or surface-absorbed.^113^ Nanocapsules and nanospheres can further be classified into polymersomes, micelles, and dendrimers.

Polymersomes, also known as engineered polymer vesicles, are composed of amphiphilic block copolymers.^114^ Self-assembly of amphiphilic copolymers forms hollow spheres with an aqueous core surrounded by a bilayer membrane. Similar to liposomes, polymersomes exhibit amphiphilicity, but, they have larger molecular weights and structures, higher stability, and greater cargo-retention efficiencies.^115^ Multitudinous polymers, such as poly(ethylene glycol) (PEG) and poly(ethylene oxide) (PEO) are commonly used in polymersome formation. Sizes, physicochemical properties, morphologies, surface activities, and stimuli-responsiveness of polymersomes can be customized by adjusting the ratio of amphiphilic copolymers.^115^ Therefore, polymersomes are ideal carriers for the delivery of diagnostic and therapeutic molecules.^116^

Polymeric micelles are formed by self-assembly of amphiphilic block copolymers in aqueous environments.^117^ These nanoparticles are nanospheres with a hydrophilic core and a hydrophobic shell. The core of micelles exhibits the ability to stabilize and solubilize poorly soluble compounds, while the coating can be loaded with hydrophilic drugs.^118^ Some polymers that are commonly copolymerized for micelles include PEG and polylactides (PLA). Polymeric micelles, whose average diameters range from 10 to 100 nm, possess several advantages, such as high structural stability, high water solubility, low toxicity, and separated functionality.^119,120^ Besides, these micelles can carry diverse compounds and provide longer circulation time as well as better accumulation.^121^

Dendrimers are highly branched nanoparticles with complex three-dimensional structures. They are composed of multiple internal repeating units covalently linked to the nucleus (called generations) and usually possess multiple functional groups on the exterior. Monodispersity, nanosize, bioavailability, solubility, biocompatibility, permeability, interactions with membranes, and interior cavities of dendrimers make them very attractive in biomedical applications, specifically as drug vesicles.^122–125^ They can carry various cargos, such as nucleic acids and small molecules.^126^ Active functional groups on the periphery of dendrimers can conjugate bioactive molecules and imaging agents to the surface, while drugs can be loaded on the inside.^127^

### Biomimetic nanoparticles

**Fig. 3:**
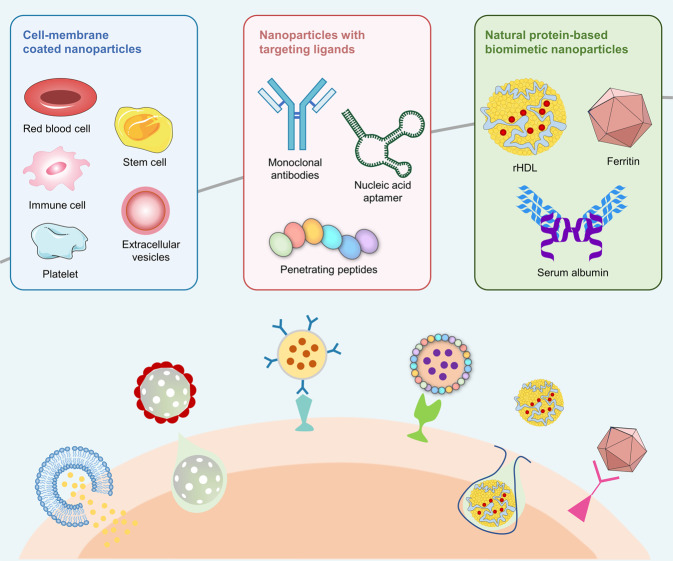
Schematic illustration of biomimetic nanoparticles. It mainly includes cell-membrane-coated nanoparticles, nanoparticles with targeting ligands, and natural protein-based biomimetic nanoparticles. rHDL reconstituted high-density lipoprotein

Biomimetic nanoparticles are formed by integrating different biomaterials onto surfaces of nanoparticles, which enables them to mimic the biological characteristics and roles of native cells.^128^ Compared to traditional nanoparticles, biomimetic nanoparticles are characterized by low immune responses, long-term blood circulation, high target specificity, and excellent biocompatibility, which can improve the specificity and biocompatibility of drugs in ideal lesions.^129^ Three principal types of biomimetic nanoparticles, including cell-membrane coated nanoparticles, nanoparticles with targeting ligands, and natural protein-based biomimetic nanoparticles have been extensively studied, especially in vascular aging and related diseases (Fig. 3).

Cell membrane-coated nanoparticles have received tremendous attention. A variety of cell membranes, such as those from red blood cells (RBCs),^34^ platelets,^130^ immune cells,^131,132^ and extracellular vesicles (EVs)^133^ have been utilized for encapsulating nanoparticles. It has been reported that RBCs membrane-coated nanoparticles could evade immune clearance and maintained a long circulation time.^34^ Human platelet membrane-cloaked polymeric nanoparticles exhibit platelet-associated immunomodulatory and antigen adhesion functions. Compared to uncoated nanoparticles, platelet membrane-enclosed nanoparticles showed decreased uptake by macrophage-like cells and increased therapeutic efficacies.^130^ Cheng et al. prepared macrophage membrane-coated biomimetic reactive oxygen species (ROS)-responsive nanoparticles for atherosclerosis treatment. Macrophage membranes avoid the clearance of nanoparticles by the reticuloendothelial system and inhibit local inflammation by sequestering pro-inflammatory cytokines.^131^ EVs are secreted by almost all cell types and contain various cargos, such as proteins, nucleic acids, and lipids.^134^ Expressions of CD47 on EV membranes offer immune evasion abilities. EVs play vital roles in vascular aging and related diseases.^135^

Nanoparticles with targeting ligands have been developed to enhance their accumulation in specific disease lesions and to improve their therapeutic efficacies. Ligands such as antibodies,^136^ antibody fragments,^137^ peptides,^138^ and other small molecules have been used to develop targeted functionalized nanoparticles. Expressions of intercellular adhesion molecule-1 (ICAM-1) by ECs and VSMCs are upregulated in vascular aging-related diseases, such as atherosclerosis, myocardial infarction (MI), and stroke. Anti-ICAM-1 antibody-conjugated nanoparticles have the potential for non-invasive molecular imaging of inflammation and targeted drug delivery.^136,139^ Nanoparticles functionalized with human single-chain variable fragment (scFv) antibodies have been assessed for multimodal molecular imaging in ApoE^−/−^ mouse models.^137^ Xu et al. constructed VHPKQHR peptide-modified MSNs as magnetic resonance (MR) contrast agents for monitoring atherosclerosis lesions.^138^

Proteins are primary components in the human body and are implicated in a broad range of cellular processes. Their superb structural integrity and multifaceted functions enable them to be easily reprogrammed and modified. Due to their outstanding versatility and biocompatibility, the ability of protein-based biomimetic nanoparticles, such as reconstituted high-density lipoprotein (rHDL) nanoparticles,^140^ ferritin protein cages,^141^ and albumin-fabricated nanoparticles^142^ as targeted drug delivery vehicles have been widely researched. Sequential administration of apoA-I-rHDL nanoparticles promoted the targeting of atherosclerotic lesions and improved prognosis in triple-cell 2D-atheroma plaque models.^143^

## Vascular aging and related diseases

Vascular aging, defined as the functional and structural alterations of the vasculature, is characterized by enlarged lumens, increased vascular stiffness, and decreased vascular elasticity.^144^ Aging is a risk factor for vascular diseases. Vascular aging can lead to progressive deterioration of organ functions.^145^

### Mechanisms of vascular aging

**Fig. 4:**
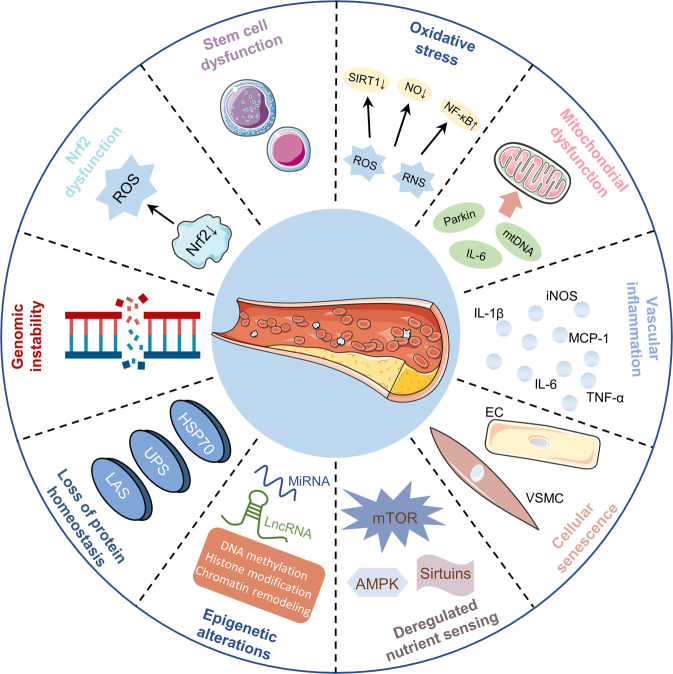
Mechanisms of vascular aging. A broad range of molecular and cellular events, including oxidative stress, mitochondrial dysfunction, vascular inflammation, cellular senescence, epigenetic alterations, genomic instability, impaired resistance to molecular stressors, deregulated nutrient sensing, loss of protein homeostasis, and stem cell dysfunction are involved in the pathology of vascular aging. This figure was created with the aid of Servier Medical Art (https://smart.servier.com/). ROS reactive oxygen species, RNS reactive nitrogen species, SIRT1 sirtuin 1, NO nitric oxide, NF-κB nuclear factor-kappaB, IL-6 interleukin-6, mtDNA mitochondrial DNA, iNOS inducible nitric oxide synthase, MCP-1 monocyte chemotactic protein-1, TNF-α tumor necrosis factor alpha, EC endothelial cell, VSMC vascular smooth muscle cell, mTOR mechanistic/mammalian target of rapamycin, AMPK adenosine monophosphate protein kinase, miRNA microRNA, lncRNA long non-coding RNA, UPS ubiquitin-proteasome system, LAS lysosome-autophagy system, Nrf2 nuclear factor erythroid 2-related factor 2

To develop effective therapeutic approaches for improving vascular aging and preventing age-related vascular pathologies, it is necessary to establish the molecular and cellular alterations during vascular aging (Fig. 4). A broad range of molecular and cellular events, including oxidative stress, mitochondrial dysfunction, vascular inflammation, cellular senescence, epigenetic alterations, genomic instability, impaired resistance to molecular stressors, deregulated nutrient sensing, loss of protein homeostasis, and stem cell dysfunctions are involved in the pathology of vascular aging.^2^

#### Oxidative stress

Oxidative stress refers to excess production of free radicals and reactive metabolites in response to various harmful stimuli, resulting in imbalances between pro-oxidation and anti-oxidation systems, leading to cell and tissue damage.^146,147^ Oxidative stress is key in vascular aging and is also a central consequence of vascular aging.^2^ The production of ROS and reactive nitrogen species (RNS) increases with vasculature aging.^148,149^ ROS production in vascular walls is predominantly due to the actions of NADPH oxidase (NOX), xanthine oxidase, and uncoupled endothelial synthase (eNOS).^150^ Elevated oxidative stress levels lead to endothelial dysfunction by decreasing the bioavailability of nitric oxide (NO), impairing vasodilation, and altering endothelial phenotypes.^151^ NO has anti-thrombotic, anti-inflammatory, anti-leukocyte adhesion, and anti-intima proliferation roles, which are essential for regulating blood flow and vasodilation.^152^ Age-related endothelium-dependent dilation downregulation is tightly associated with endothelial oxidative stress. Elevated NOX and nuclear factor-kappa B (NF-κB) levels are vital sources of oxidative stress in ECs.^153^ Multiple vascular risk factors, such as hypercholesterolemia, hypoxia, diabetes mellitus, hypertension, obesity, and smoking can increase ROS levels and decrease the generation of endothelial NO.^3,150,154^ Elevated ROS levels reduce NO bioavailability through the formation of toxic peroxynitrite. Besides, peroxynitrite uncouples eNOS, leading to increased oxidative stress and decreased eNOS-derived NO.^155^ ROS and RNS have also been shown to promote the proliferation and migration of VSMCs, leading to vascular stiffness and cell senescence.^156^ Excess ROS and oxidative stress triggers vascular remodeling through inducing vascular inflammation, vascular cell impairment, matrix metalloproteinases (MMPs) activation, lipid peroxidation, and extracellular matrix (ECM) deposition.^157^ Numerous lines of evidence suggested that oxidative stress and ROS are involved in the initiation and progression of vascular aging and related diseases, such as atherosclerosis, hypertension, vascular restenosis, ischemic stroke, and cerebral hemorrhages.^9,158–160^

#### Mitochondrial dysfunction

Mitochondrial dysfunction is a hallmark of aging and a vital mechanism of vascular aging.^161^ Aged vasculature is associated with elevated mitophagy protein Parkin levels, causing mitochondrial dysfunction and enhanced mitophagy. Additionally, the aged vascular system induces increased expressions of inflammatory cytokines, including interleukin (IL)-6, leading to mitochondrial damage. In turn, mitochondrial damage promotes IL-6 generation by activating the toll-like receptor 9 (TLR9)-MyD88 signaling pathway.^162^ Arterial mitochondrial respiration significantly decreases with age. Suppression of mitochondrial functions and dysregulated mitochondrial DNA integrity is directly correlated with vascular aging.^163^ Excess ROS generation by the mitochondria is another critical mechanism of vascular aging.^164^ Mitochondrial ROS can be generated via the inhibition of manganese superoxide dismutase (MnSOD), peroxynitrite-mediated nitration, downregulation of p66, and reduction of cellular glutathione.^2^ Mitochondrial-derived ROS contributes to pro-inflammatory phenotypic alterations in the aged vascular systems via NF-κB activation.^165^ Mitochondria-related oxidative stress aggravated ECs and VSMCs senescence by activating the Akt signaling pathway and the NF-κB/NOX1 axis, respectively.^166,167^ Besides, mitochondrial-related ROS induces vascular cell apoptosis in a Bcl-2-dependent manner.^168^ Thus, mitochondrial-derived ROS accelerates vascular aging by promoting vascular inflammation, enhancing cell senescence, and inducing apoptosis.

#### Vascular inflammation

Vascular aging is a chronic, sterile, low-grade inflammation process that is tightly associated with endothelial dysfunction and arterial stiffness. Converging evidence has implicated that the gene expression profiles of ECs and VSMCs have been associated with pro-inflammatory alterations in aged animal models.^169^ Vascular aging-related inflammation is characterized by overexpressed inflammatory cytokines, including C-reactive protein (CRP), vascular cell adhesion molecule-1 (VCAM-1), adhesion molecules, and pro-inflammatory cytokines.^170^ Vascular inflammation mechanisms are multifaceted. Oxidative stress induces chronic vascular inflammation by activating several transcription factors, such as NF-κB, AP-1, and peroxisome proliferator-activated receptor-γ (PPAR-γ).^171^ The ROS-sensitive NF-κB signaling pathway is critical in aging-related vascular inflammation.^153^ In aged vasculatures, oxidative stress and vascular inflammation act in a vicious cycle.^170^ Sirtuin 1, an anti-aging molecule, is downregulated in aged vascular tissues. Suppressed sirtuin1 levels in ECs and VSMCs promote vascular aging through multiple mechanisms, including oxidative stress, vascular inflammation, cellular senescence, reduced NO expressions, and impaired autophagy.^172^ ECs senescence is positively correlated with levels of various inflammatory cytokines and chemokines, including IL-6, IL-1α, and monocyte chemotactic protein (MCP-1).^173^ In addition, elevated oxidative stress and inflammation levels and reduced NO bioavailability can alter transforming growth factor-β (TGF-β) and MMPs expressions, thereby promoting vascular aging. The vascular inflammation microenvironment stimulates vascular aging-related disease development by inducing endothelial dysfunctions, cellular metabolism impairments, and cell apoptosis.^2^

#### Cellular senescence

Cell senescence, a cell aging process that is initiated by responses to various endogenous and exogenous stressors, involves various unique phenotypic alterations in cells.^166^ ECs and VSMCs, predominant cell types in the vasculature, are involved in the formation of vascular endothelium and vascular media layer, respectively. ECs are crucial in controlling vascular constriction and relaxation, blood fluidity, angiogenesis, inflammation, and immune responses.^174^ The shift of ECs towards pro-inflammatory states, pro-thrombotic phenotypes, and decreased vascular tones is collectively termed endothelial dysfunction.^151,175^ VSMCs play important roles in the regulation of blood flow and vascular tension. Under the pathological conditions, VSMCs phenotypes transform from quiescent to proliferative and migratory.^176^ VSMCs aging-induced calcification and stiffening are closely correlated with diverse vascular disorders.^177^ Functional and structural alterations of ECs and VSMCs are critical features of vascular aging. ECs and VSMCs have a great untapped potential as therapeutic targets in vascular aging.

#### Epigenetic alterations

Epigenetic alterations are involved in the development of vascular aging by modulating the function and phenotype of ECs and VSMCs.^178^ Epigenetics, including DNA/RNA methylation, histone modifications, microRNAs (miRNAs), and long non-coding RNAs (lncRNAs) exhibited a broad range of roles in vascular aging progression.^135,179,180^ In mammals, DNA methylation involves the transfer of a methyl group to the C5 position of cytosine. DNA methylation recruited gene repression proteins or suppressed transcription factors bind DNA, thereby modulating gene expressions.^181^ During vascular aging, DNA methylation patterns within vascular cells are altered.^179^ RNA methylation occurs in all stages of the RNA lifecycle, including RNA processing, nuclear export, translation regulation to RNA degradation, implying that it is an essential internal modification of RNA metabolism. It has been recognized that RNA methylation, especially N6-methyladenosine, shows a regulatory impact on DNA damage, immunity, cell growth, apoptosis, and aging.^182^ Histone acetylation is regulated by histone deacetylases and histone acetyltransferases.^183^ Suppressed expressions or activities of class III histone deacetylases have a role in vascular aging.^184^ MiRNAs, with approximately 22 nucleotides, negatively regulate gene expressions by preventing translation or by promoting gene degradation at the post-transcriptional level.^185^ LncRNAs, over 200 nucleotides in length, are mainly transcribed by RNA polymerase II. LncRNAs regulate gene expressions by introducing chromatin-modifying enzymes at specific genomic sites, separating transcription factors from genomic targets, or acting as miRNA sponges.^185,186^ MiRNAs and lncRNAs have significant effects on vascular aging and related disorders.^180,187^

### Vascular aging-related diseases

Vascular aging is a strong predictor of mortality from multiple vascular disorders, including cardiovascular diseases, cerebrovascular diseases, and chronic kidney diseases. Vascular aging-related diseases affect health span and potential life span in mammals.^170^

#### Cardiovascular diseases

According to the World Heart Federation, annually, cardiovascular diseases cause 17.3 million deaths.^188^ Diagnostic, treatment, and nursing costs are rapidly increasing. With an expanding elderly population, cardiovascular diseases are projected to become the leading global cause of morbidity and mortality.^189^ It is estimated that annual deaths from cardiovascular diseases, especially heart disease and stroke will account for more than 23.3 million people by 2030.^190^ Hypertension, which is a key player in various cardiovascular diseases, such as atherosclerosis, heart failure, and ischemia, is highly attributed to the increasing mortality rates from cardiovascular diseases. In line with a report disclosed in 2015, it was documented that globally, about 1.13 billion people suffer from hypertension,^191^ which is projected to rise to 1.60 billion people by 2025.^192^ Atherosclerosis is a chronic inflammatory condition that is highly involved in the development of cardiovascular diseases. Pathological mechanisms of atherosclerosis are intricacy, including vascular cell dysfunction, chronic inflammatory responses, and elevated lipoprotein cholesterol concentrations. Of all the triggers, vascular aging remains the strongest connection with the prevalence of atherosclerosis. A plausible explanation is that vascular aging-associated mechanisms play crucial roles in the pathophysiology of atherosclerosis.^193^ Restenosis, predominantly caused by intimal hyperplasia, is directly correlated with vascular remodeling and ECM deposition. It occurs as a serious complication after angioplasty.^194^ Coronary arterial disease (CAD) refers to the formation of atherosclerotic plaques in the vessels that supply nutrients and oxygen to the heart.^195^ Epidemiological studies of CAD support that age, obesity, hyperlipidaemia, diabetes, hypertension, and smoking increase the risk of MI. Every 34 seconds, an American experiences a MI or cardiac death.^196^ MI, refers to ischemic necrosis of cardiomyocytes, is one of the main causes of heart failure, resulting in irreversible loss of cardiomyocytes and cardiac function deterioration.^197^ The current therapeutic options for MI are generally ineffective as they principally aim at ameliorating progression and relieving symptoms, rather than repairing the damaged myocardium. Heart failure is a systemic, multifactorial disease, affecting around 1% to 2% of the adult population.^198^ Notably, heart failure is highly prevalent among the elderly, with its prevalence among 65- to 70-year old increasing steadily from 4.3% in 2012 to 8.5% in 2030. It is a major clinical and public health problem.^199^

#### Cerebrovascular diseases

After cardiovascular diseases, cerebrovascular diseases are the second leading cause of death worldwide. Vascular aging-related cerebrovascular diseases, including ischemic stroke, intracerebral hemorrhage (ICH), and vascular dementia, represent a massive burden on economic and social health.^200^ Therefore, there is an urgent need to develop effective prevention and treatment options for these conditions. Ischemic stroke, with over 795,000 annual cases, accounts for more than 80% of cerebrovascular diseases. It is the main cause of long-term disability.^201^ After ischemic stroke, ICH is the second most common subtype of stroke, accounting for 10% to 20% of all strokes. With increasing life expectancy, the health and economic burden of ICH is also increasing.^202^ Vascular dementia, a cognitive decline arising from vascular lesions, is a common cause of dementia after Alzheimer's disease, accounting for 15% of cases. However, there are no licensed therapeutic strategies for vascular dementia.^203^

#### Chronic kidney disease

Chronic kidney disease is defined as a structural or functional abnormality of the kidney that lasts for more than three months.^204^ Globally, it is an irreversible and progressive disease with a high prevalence of 13.4% (11.7–15.1%). It has been identified as a major public health problem that is associated with high cardiovascular risks.^205,206^

## Nanoparticle-based diagnostic strategies for vascular aging-related diseases

**Fig. 5:**
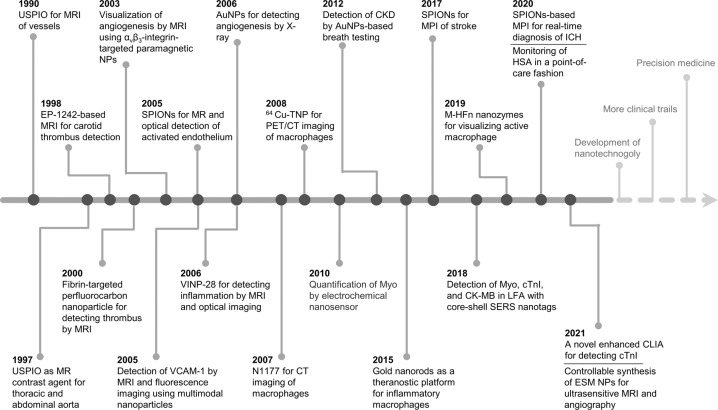
Historical timeline of nanoparticles used in the diagnosis of vascular aging-related diseases. This timeline scheme was made using the Web of Science database. Key discoveries are highlighted. USPIO ultrasmall superparamagnetic iron oxide particles, MR magnetic resonance, MPI magnetic particle imaging, SPIONs superparamagnetic iron oxide nanoparticles, NPs nanoparticles, VCAM-1 vascular cell adhesion molecule-1, CT computed tomography, PET positron emission tomography, Myo myoglobin, CKD chronic kidney disease, AuNPs gold nanoparticles, cTnI cardiac troponin I, CK-MB creatine kinase-muscle/brain test, LFA lateral flow assay, SERS surface-enhanced Raman scattering, M-HFn magnetoferritin nanoparticles, ICH intracerebral hemorrhage, HSA human serum albumin, CLIA chemiluminescent immunoassay, ESM exceptionally small-sized superparamagnetic magnetite

Global life expectancy is increasing, with about one-fifth of the world's population estimated to be above 65 years by 2030.^207^ Age is a vital risk factor affecting vascular homeostasis.^208^ With the aging population, the prevalence of vascular diseases is exponentially increasing, becoming a social and economic burden. Due to the high mortality and disability of vascular aging-related disorders, early diagnosis shows beneficial effects in delaying the progression and improving the prognosis of vascular disorders.^209^ Currently, vascular disease diagnosis is based on the detection of biomarker levels and angiography.^8^ Most diagnostic techniques are costly, with low sensitivity. Therefore, the development of cheaper, faster, and more efficient methods for early diagnosis is of great necessity. Applications of nanoparticles in the diagnosis of vascular aging and related diseases have been under exploration with striking outcomes (Fig. 5).

### Biosensors

Biomarkers are defined as characteristic indices that can objectively reflect and evaluate normal physiological processes, pathophysiological processes, or drug treatment responses.^210^ Detection of specific biomarkers for vascular aging-related diseases, including nucleic acids (DNA and RNA), proteins, and antibodies, is essential for understanding their role in early diagnosis, treatment, and prognosis of disease. However, due to various technical difficulties existing in the current detection of biomarkers, their full potential has not been realized. Primarily, biomarkers are typically present at extremely low concentrations and are always mixed with other substances, which inhibits their detection. Secondly, assaying for biomarkers at very low concentrations is challenging and time-consuming in many cases.^211^ In addition, for clinical diagnostic applications, multiple biomarker detection methods, including radioimmunoassay,^212^ gel electrophoresis,^213^ enzyme-linked immunosorbent assay (ELISA),^214^ meso-scale discovery (MSD),^215^ high-performance liquid chromatography (HPLC),^216^ protein microarrays,^217^ and quantitative reverse transcription PCR (RT-PCR),^218^ are suffer from the same drawbacks such as low sensitivity, poor accuracy, and weak specificity. Compared to traditional detection techniques, nanoparticle-based biosensors have desirable advantages of easy operation, high sensitivity, excellent stability, good specificity, fast response, and cost-effective analysis.^219^ Therefore, nanoparticle-based biosensors have potential applications for selective, ultra-sensitive, and robust detection of these low-abundance biomarkers in body fluids (plasma, serum, and urinary).^209^ In the past two decades, due to their optical, electrochemical, and intrinsic magnetic properties, magnetic nanoparticles, especially AuNPs, have been identified as ideal nanomaterials in biosensing (Table 2).

**Table 2:** Nanoparticles-based biosensors in vascular aging-related diseases

Diseases	Nanoparticles	Biomarkers	Detection Limit	Technique	Ref(s)
Atherosclerosis	AuNPs	POVPC PONPC	0.17 nM, 0.44 nM	LC-ESI-MS/MS	^220^
Hypertension	AuNPs	ACE gene	1nM	EIS	^226^
	AuNPs	Cortisol	0.342 μg/dL	CL-LFA	^229^
	QD	Renin	25 pM	TIRF microscopy	^228^
MI	TiO_2_ NPs	Myoglobin	0.22 ng/mL	Electrochemical detection	^230^
	PEI-AuNPs	Myoglobin	6.29 ng/mL	Electrochemical detection	^231^
	HsGDY@NDs	cTnI, Myoglobin	9.04 fg/mL, 6.29 fg/mL	Impedimetric aptasensing	^232^
	GNRs	cTnI	10 ng/mL	Surface plasmon resonance	^572^
	AuNPs	cTnI, copeptin, H-FABP	0.3 pg/mL, 0.4 pg/mL, 0.06 pg/mL	Chemiluminescence	^233^
	GNVs	cTnT, CK-MB, NT-proBNP,	7.8 pg/mL, 910 pg/mL, 70 pg/mL	EFISA	^234^
	AuNPs	cTnI	5.7 ng/L	Digital immunoassay	^573^
	GQDs-AuNPs	cTnI	0.5 pg/mL	Enzyme-free electrochemical detection	^574^
	GO-AuNPs	cTnI	0.05 ng/mL	Electrochemical immunoassay	^575^
	AuNPs	cTnI	16 pg/mL	Electrochemical detection	^576^
	ABEI-AuNPs	cTnI	2 pg/mL	Electrochemiluminescence immunoassay	^577^
	AuNPs	cTnT	5 ng/mL	Surface plasmon resonance	^578^
	AuNPs	Hs-cTnT	6.2 ng/L	Digital immunoassay	^579^
	GNSs	Exosomal HIF-1α	0.2 ng/L	Colorimetric determination	^580^
	Ag/Au nanosphere	MiRNA-133a	0.306 fM	Surface plasmon resonance	^581^
Ischemic Stroke	Graphene	MMP-2	17 ng/mL	Tracking spectral shift	^245^
	AuNPs	CRP	4.6 pg/mL	ECL-LFI	^244^
	Sandwich NPs	NSE	0.86 ng/mL	Immunoassay	^246^
ICH	Gold nanostars	GFAP	0.54 fg/mL	Immunoassay	^252^
CKD	AuNPs	Creatinine	13.7 mg/L	Surface plasmon resonance	^262^

*AuNPs* gold nanoparticles, *POVPC* 1-palmitoyl-2-(5′-oxovaleroyl)-sn-glycero-3-phosphocholine, *PONPC* 1-palmitoyl-2-(9′-oxononanoyl)-sn-glycero-3-phosphocholine, *LC-ESI-MS/MS* liquid chromatography-electrospray ionization-tandem mass spectrometry, *ACE* Angiotensin-converter enzyme, *EIS* electrochemical impedance spectroscopy, *CL-LFA* chemiluminescence-based lateral flow assay, *QD* quantum dot, *TIRF* total internal reflection fluorescence, *MI* myocardial infarction, *TiO*_*2*_ *NPs* titanium oxide nanoparticles, *PEI* polyethylenimine, *cTnI* cardiac troponin I, *HsGDY@NDs* heteronanostructure of nanodiamonds and hydrogen-substituted graphdiyne, *GNRs* gold nanorods, *GNVs* gold nano-vesicles, *H-FABP* heart-type fatty acid-binding protein, *NT-proBNP* N-terminal prohormone of brain natriuretic peptide, *CK-MB* kinase-muscle/brain test, *cTnT* cardiac muscle troponin, *EFISA* enzyme-free immunosorbent assay, *GQDs* graphene quantum dots, *GO* graphene oxide, *IONs* iron oxide nanoparticles, *hs-cTnT* high-sensitivity cardiac troponin T, *GNSs* Gold nanospheres, *HIF-1α* hypoxia-inducible factor-1 alpha, *MMP-2* matrix metalloproteinase 2, *CRP* C-reactive protein, *NSE* neuron-specific enolase, *ICH* intracerebral hemorrhage, *GFAP* glial fibrillary acidic protein, *CKD* chronic kidney disease

#### Cardiac biomarkers detection

It has been reported that biomarkers in body fluids are potentially effective and sensitive signals for early diagnosis of vascular aging-related diseases. Early detection of cardiac biomarkers for individuals at a high risk of vascular aging-related cardiovascular diseases, including atherosclerosis, hypertension, and MI can reduce the risk of death. To date, the detection of cardiac biomarkers is predominantly based on the traditional ELISA technique, which is a time-consuming and labor-intensive work. Thus, the development of a uniform, rapid, and convenient detection strategy for cardiovascular events is of great significance. As biosensors, nanoparticles have attracted tremendous attention in detecting cardiac biomarkers.

Oxidized low-density lipoproteins (ox-LDLs), such as oxidized phospholipids (oxPLs),^220^ oxidized phosphatidylcholines (oxPCs),^221^ and malondialdehyde-modified low-density lipoprotein (MDA-LDL),^222^ play an important role in the initiation and progression of atherosclerosis, and are risk biomarkers for oxidative stress. However, their abundance in plasma is low. AuNPs-based bioanalysis offers a sensitive and fast detection of oxidative stress lipid biomarker screening. Additionally, the inflammatory biomarker, ICAM-1, is also an effective signal for atherosclerosis screening. Surface-enhanced Raman scattering (SERS) probe gold nanorods (GNRs) are sensitive options for early detection of ICAM-1 in macrophages.^223^ In addition, compelling evidence indicates in-negligible roles of AuNPs in the field of hypertension identification. Overexpressed epithelial sodium channel (ENaC) in membrane platelets is strongly associated with arterial hypertension.^224^ García-Rubio et al. proposed a new diagnostic tool for distinguishing normal blood pressure from hypertension by conjugating AuNP with an anti-ENaC. The indirect immunofluorescence detection assay revealed a tendency of fluorescence signals and increased fluorescence intensity in platelets treated with anti-ENaC-conjugated AuNPs.^225^ In view of the relationship between systemic arterial hypertension (SAH) and hypertension, early SAH diagnosis is of great significance. Geno-sensors, which are based on nanoparticles, are applied to diagnose genetic disorders by detecting specific DNA sequences. Rolim et al. developed an AuNPs-containing geno-sensor for the detection of SAH polymorphisms in intron 16 of the ACE gene.^226^ Cortisol and renin are hypertension biomarkers.^227,228^ A portable chemiluminescence-based lateral flow assay platform was synthesized by conjugating AuNPs with the anti-cortisol and anti-horseradish peroxidase antibodies, which can be used for serum cortisol detection.^229^ Besides, Long et al. employed a Cy5-labeled and streptavidin-coated QD probes to detect plasma renin activities, which are tightly associated with hypertension and congestive heart failure.^228^

Myoglobin, cardiac troponin I (cTnI), cardiac troponin T (cTnT), heart-type fatty acid-binding protein (H-FABP), and creatine kinase-muscle/brain test (CK-MB) are potential MI biomarkers. Early detection of these biomarkers can reduce the risk of death. A label-free electrochemical biosensor can be used for the effective and sensitive detection of myoglobin levels and the assessment of MI phases.^230^ Monoclonal anti-myoglobin antibody-coated polyethylenimine (PEI)-AuNPs have the ability for quantitative detection of myoglobin, with a detection range from 9.96 ng/mL to 72.8 ng/mL and a detection limit of 6.29 ng/mL.^231^ Anodiamonds and hydrogen-substituted graphdiyne mixture (HsGDY@NDs) have excellent sensing performance for myoglobin and cTnI detection, with low detection limits of 9.04 fg/mL and 6.29 fg/mL, respectively.^232^ As amplified capture probes and amplified signal probes, functionalized AuNPs have been employed for simultaneous detection of cTnI, copeptin, and H-FABP. This method exhibited an ultra-wide detection range for cTnI (0.5 pg/mL to 1 μg/mL), copeptin (1 pg/mL to 1 mg/mL), and H-FABP (0.1 pg/mL to 1 μg/mL). Besides, detection limits of the present method for cTnI, copeptini, and H-FABP were established to be 0.3 pg/mL, 0.4 pg/mL, and 0.06 pg/mL, respectively.^233^ The enzyme-free immunosorbent assay (EFISA) of three-dimensional gold nanovesicles integrated with three allochroic agents could be applied for the detection of cTnT, CK-MB, and N-terminal prohormone brain natriuretic peptide (NT-proBNP).^234^

Brain natriuretic peptide (BNP),^235^ NT-proBNP,^236^ CRP,^237^ antigen galectin-3 (GL-3),^238^ and miRNA-21,^239^ have been recognized as critical cardiac biomarkers for the diagnosis and prognosis of heart failure. Lei et al. developed a platinum nanoparticles-modified reduced graphene oxide biosensor for label-free and high sensitive detection of BNP in whole blood. It allows a low detection limit of 100 fM.^240^ Silver nanoparticle-based microfluidic biosensors have the potential for sensitive quantification of NT-proBNP, with a limit of detection of 0.57 ng/mL.^236^ As sensor platforms, AuNPs-decorated graphitic carbon nitride nanosheets were used for antigen GL-3 detection in plasma samples. They exhibited a wide linearity range of 0.0001 ng/mL to 20.0 ng/mL and a low detection limit of 0.025 pg/mL.^238^ A carbon nanodot-based electronic chemiluminescence biosensor was developed for selective and sensitive detection of miRNA-21 in serum samples, with a linear response concentration of up to 100.0 pM and a detection limit of 0.721 fM.^239^

Nanoparticles such as AuNPs, graphene, and carbon dots are critical protagonists in different types of biosensors to enable ultra-sensitive and multiple detection of cardiac biomarkers, including myoglobin, cTnI, cTnT, CK-MB, H-FABP, exosomes, and miRNAs.^241–243^

#### Brain biomarkers detection

Screening of cerebrovascular disease-related biomarkers is indispensable to improving individualized treatment and reducing mortality. Nevertheless, there is still a lack of safe, sensitive, and rapid diagnostic strategies for vascular aging-related cerebrovascular diseases. Nanoparticles-based optical and electrochemical biosensors have been extensively investigated in the field of brain biomarkers detection.^219^

Ischemic stroke accounts for more than 80% of cerebrovascular diseases. However, the diagnosis of acute-phase stroke is challenging. Biologically, CRP,^244^ MMPs,^245^ neuron-specific enolase (NSE),^246^ and S-100B^247^ are associated with ischemic stroke. A full-range CRP test is critical for identifying patients who require intensive treatment or close follow-up after ischemic stroke or MI. Ru(bpy)_3_^2+^-labeled AuNPs exhibited rapid and high sensitivity in detecting CRP levels in spiked serum, with a wide detection range of 0.01–1000 ng/mL and a detection limit of 4.6 pg/mL within 15 min. They have a great potential for detecting CRP levels at point-of-care diagnostics.^244^ In addition, MMPs, especially MMP-2,^248^ MMP-7,^249^ and MMP-9,^250^ are highly associated with stroke. Thus, their effective and sensitive screening is pivotal for stroke diagnosis. A class of optical interference-free SERS nanotags was employed for convenient and multiple detection of relevant biomarkers.^251^ For instance, Lin et al. prepared a monolayer graphene-ruthenium carbonyl cluster-based biosensor for the quantitative detection of MMP-2, with a detection limit of 17 ng/mL.^245^ Additionally, NSE and S-100B proteins have been found to be elevated in patients with ischemic brain injury.^247^ Paper-based lateral flow strip (PLFS) based on SERS was successfully used for NSE detection, with a detection limit of 0.86 ng/mL.^246^

Early detection of ICH biomarkers, such as glial fibrillary acidic protein (GFAP), has a high beneficial effect in early diagnosis and informing clinical decisions. Based on gold nanostars, Zhao et al. developed a SERS-based immunoassay for detecting GFAP, with a broad range of 1 pg/mL to 1 μg/mL and a detection limit of 0.54 fg/mL.^252^ Additionally, there are particularly strong data indicating that plasma tau protein levels in patients with vascular dementia are significantly higher than those in healthy subjects. Antibody-functionalized magnetic nanoparticles can be employed for the detection of total tau proteins in human plasma via an immunomagnetic reduction method.^253^

Clinically, urine analysis has long been used for monitoring health and disease during medical examinations.^254–256^ Synthetic biomarkers may be developed to remotely sense vascular disorders using urine samples, with potential applications in point-of-care diagnostics. Thrombin is essential for the formation of thrombosis, a life-threatening condition related to atherosclerosis and stroke. To overcome the low specificity of traditional detection techniques and the inability to detect thrombin activity, Lin et al. designed and combined a thrombin-sensitive peptide substrate to the surface of iron oxide nanoworms. After intravenous infusion, these synthetic biomarkers were able to monitor coagulation and thrombin activities in the vasculature, and release ligand encoded reporters into urine.^254^

#### Kidney biomarkers detection

Numerous lines of evidence demonstrated that nanoparticles can be applied for the detection of kidney biomarkers, such as creatinine,^257^ cystatin C (CysC),^258^ uric acid (UA),^259^ human serum albumin (HSA),^260^ and neutrophil gelatinase-associated lipocalin (NGAL).^261^ Serum or urinary creatinine concentrations are essential and indispensable clinical analyses for renal function assessment. Ortiz-Gómez et al. used luminescence spectroscopy-based europium-doped amorphous calcium phosphate nanoparticles to assess creatinine levels in a sensitive, selective, and stable manner.^257^ Label-free AuNPs have the potential for detecting human urinary creatinine. This approach is suitable for creatinine concentration ranges of 15 mg/L to 40 mg/L, with a low detection limit of 13.7 mg/L.^262^

### Bioimaging

To date, another commonplace diagnostic technique applied in clinical settings is angiography, including invasive imaging approaches, such as intravascular ultrasound (IVUS), optical coherence tomography (OCT), near-infrared spectroscopy (NIRS) and non-invasive imaging methods such as computed tomography (CT), computed tomographic coronary angiography (CTCA), MRI, positron emission tomography (PET), and single-photon emission computed tomography (SPECT). Grayscale IVUS can be used for evaluating vessel wall dimensions, phenotypic characteristics, and severity of atherosclerotic lesions.^263^ A prospective study (NCT00180466) reported that IVUS failed to visualize the entire coronary tree and assessed only 53% of the lesions that caused adverse cardiovascular events during a median follow-up time of 3.4 years.^264^ Besides, IVUS-based modalities may suffer several technical limitations, such as spatial resolution and operator-dependent parameters.^265^ Compared to IVUS, OCT offers small inexpensive designs, faster data acquisition rates, a higher resolution (10–20 μm), and the visualization of smaller vessels.^266^ Principal constraints of OCT include the attenuation of OCT optical beams and the low penetration depths of 2–3 mm, resulting in unclear visualization of vessel walls and preventing plaque burden assessment, respectively.^267^ It is a suitable approach for quantitative and reliable estimation of lipid compositions of core plaque, however, NIRS is not capable of detailed and complete plaque morphological assessment as well as visualization and evaluation of lumen, vessel wall dimensions, and plaque burden.^268^ Additionally, the advantages of PET are its superior sensitivity and excellent quantitative efficiency, however, its limitations include exposure to radiation, high costs, and limited availability.^269^ CTCA is established as a molecular imaging model with a high specificity and outstanding predictive value, but low sensitivity.^270^ MRI allows detailed assessment of arterial wall morphological parameters, but is limited by long scanning time and is unsuitable for patients with metal instruments.^265^ Furthermore, contrast agents are essential for imaging. However, clinically frequently-used contrast agents are incapable of targeting specific organs or tissues and have some shortcomings such as weak signals, short retention time, and toxic side effects.^271^

Nanoparticles, whose sizes range from 1 to 100 nm, have the capacity to cross cell membrane and tissue barriers. During the controlled processes, nanoparticles are able to stimulate, react, and interact with target cells or tissues to produce the desired physiological responses while minimizing adverse effects. By targeting specific molecules, contrast agent distributions can accurately track vascular lesions and improve the signal intensities of different imaging modalities.^272^ As contrast agents, nanoparticles can be designed and manipulated for the visualization of typical pathological alterations in vascular aging and related diseases, such as inflammation, thrombosis, angiogenesis, and apoptosis, with a great potential to improve diagnostic efficiency and accuracy.^8^ Besides, given their high biocompatibility, magnetic nanoparticles have attracted increasing attention for molecule imaging.^273^

#### Inflammation

Macrophage infiltration is a promising biomarker for multiple pathological conditions, providing information on the stage and progression of vascular disorders, such as atherosclerosis, MI, and stroke.^274^ Researchers seeking to identify and monitor inflammatory stage alternations have targeted macrophages using nanoparticles and visualized the results via MRI.^60,275,276^ Fluorescent probes can accurately detect atherosclerosis during early developmental stages, thus have been used to rapidly evaluate the effects of anti-atherosclerosis drugs. For instance, Wang et al. developed a high brightness aggregation-induced emission nanoprobe that enables early detection of atherosclerotic plaques and screening anti-atherosclerosis drugs in a sensitive, precise, and rapid manner.^277^ Another study reported that VCAM-1-targeted nanoparticles, as MR contrast agents, were a promising strategy for the diagnosis of inflammation-related disorders.^278^ Experimental results have shown that scavenger receptors AI (SR-AI) and osteopontin (OPN) were highly expressed in intraplaque macrophages.^279,280^ SR-AI-targeted ultrasmall superparamagnetic iron oxide particles (USPIO)-based MR contrast agents accumulated in intraplaque macrophages and VSMCs, indicating that this could be a promising non-invasive molecular imaging tool for in situ detection of inflammatory plaques in atherosclerosis.^281^ Besides, OPN-specific MR and optical dual-modality probe were utilized for the non-invasive detection of vulnerable atherosclerotic plaque by targeting foamy macrophages in the cytoplasm.^282^ Besides, apoA-I mimetic peptide-modified rHDL nanoparticles represent versatile delivery platforms for Gd-based contrast agents (GBCA). Numerous studies have demonstrated that GBCA-rHDL nanoparticles not only substantially accumulated in macrophages in vitro but were also taken up by intraplaque macrophages in vivo.^283–287^ Another study found that GBCA-rHDL nanoparticles functionalized with collagen-specific EP3533 peptides improved the specific target imaging efficiency of intraplaque macrophages.^288^ IONs, especially superparamagnetic iron oxide nanoparticles (SPIONs) and USPIO, have emerged as novel cell-specific MR contrast agents and have been utilized to evaluate cellular inflammation in tissues.^289–291^ Yilmaz et al. demonstrated that a USPIO-based contrast agent achieved efficient characterization of MI predominantly through detecting infiltrating macrophages. Ischemia/reperfusion (I/R) injury is correlated to vascular inflammation.^292^ SPIONs-based imaging not only exhibited superior temporal resolution but also had an excellent capability to detect perfusion deficits in the ischemic murine brain.^293^ Additionally, ECs are critical to post-stroke inflammation, where they modulate diapedesis of leukocytes from the blood to the brain by expressing adhesion molecules, such as VCAM-1, ICAM-1, and P-selectin. Notably, these adhesion molecules act as specific targets during inflammation imaging.^294–296^

#### Thrombosis

Thrombosis plays a key role in vascular aging-related disorders and related to hypoxia and tissue infarction.^297^ Therefore, direct thrombus imaging is highly beneficial in the diagnosis and treatment of thrombosis-related diseases. For instance, researchers employed thrombin-activatable fluorescent peptide (TAP)-incorporated silica-coated AuNPs (TAP-SiO2@AuNPs) for the direct imaging of thrombus via dual micro-CT and near-infrared fluorescence (NIRF) imaging.^298^ In another study, cRGD peptide-functionalized Fe_3_O_4_-PLGA nanoparticles were found to selectively and readily accumulated on the surface of thrombosis and under ECs in an abdominal aorta thrombosis rat model.^299^ Besides, Poon et al. prepared a hybrid metal oxide-peptide amphiphile micelles called HMO-Ms that comprised either manganese oxide or iron oxide inner core and fibrin-targeting peptide amphiphiles.^300^ Results from transmission electron microscopy and dynamic light scattering indicated that HMO-Ms-based MR agents were not only highly biocompatible to human aortic ECs but were also 3- to 5-fold more efficient at binding to human thrombus compared to untargeted nanoparticles.^301^ In addition, α2-antiplasmin peptide (α2AP)-targeted perfluorocarbon nanoemulsions were sensitive for monitoring thrombosis via MRI.^302^ Furthermore, glycol chitosan AuNPs have shown promise in the diagnosis of hyperacute direct thrombus through CT imaging.^303^ Platelet activation and aggregation are the initial stages of thrombosis. EWVDV-based platelet-targeting nanoparticles exhibited high binding affinity to activated platelets and were used for ultrasonography (US) of thrombi at diverse blood flow velocities.^304^ Polydopamine-based nanoparticles significantly improved targeting efficiency for thrombus by simultaneously binding to integrin αIIbβ3 and P-selectin on activated platelets. These are beneficial for early diagnosis of thrombosis-related disorders through MR and photoacoustic (PA) dual-modality imaging.^305^

#### Angiogenesis

Angiogenesis plays an important role in the development and progression of vascular disorders, and is crucial to the formation of atherosclerotic plaque, resulting in plaque hemorrhage and vulnerability.^306,307^ Therefore, urgent development of more effective targeted molecular imaging approaches for angiogenesis is imperative to the management of these disorders. Previous studies have shown that α_ν_β_3_-integrin, a heterodimer transmembrane glycoprotein, is differentially expressed in proliferating versus quiescent ECs, while during atherosclerosis, it is expressed by multiple cell types, including ECs, VSMCs, macrophages, lymphocytes, and platelets.^308^ Increasing pieces of evidence have demonstrated that integrin α_ν_β_3_-integrin-targeted paramagnetic nanoparticles as MR contrast agents play a crucial role in the detection and quantification of angiogenesis.^309,310^ Moreover, E-selectin-based nanoparticles were also found to promote the development of MR agents for monitoring angiogenesis.^311^ In another study, vascular endothelial growth factor receptor 2 (VEGFR‑2)-targeted perfluorocarbon magnetic nanocapsules served as a US/MR dual-modality probe for visualizing atherosclerotic neovasculature.^312^ Immunohistochemical staining results revealed that natriuretic peptide clearance receptor (NPR-C) was not only upregulated in angiogenic lesions, but also colocalized in both ECs and VSMCs. Moreover, Liu et al. used a ^64^Cu-labeled C-type atrial natriuretic factor (CANF) fragment to develop a novel angiogenic high-specific-activity nanoprobe for PET imaging for the detection of NPR-C.^313^ Notably, GEBP11 peptide holds specificity and high affinity for angiogenesis, thus has emerged as a specific imaging target for the visualization of vulnerable plaques by monitoring angiogenesis. As MR and PET dual-modality imaging probes, ^68^Ga-GEBP11-IONs were applied for the visualizing angiogenesis and vulnerable plaque.^314^

#### Proliferation

Abnormal proliferation and migration of VSMCs have been implicated in the development of vascular aging-related disorders. Consequently, this phenomenon not only offers a specific target for the detection of vascular disorders but also provides a potential opportunity for generating information regarding the developmental stages and progression. Previous studies have shown that profilin-1 was upregulated in cardiovascular disorders, thus played a crucial role in modulating proliferation and migration of VSMCs.^315–317^ Researchers have used profilin-1-targeted MR and fluorescence dual-modality contrast agent (PC-IONs) for non-invasive visualization of atherosclerotic plaque development.^318^ Elastin, an ECM protein, is expressed mainly by fibroblasts and VSMCs. Notably, an elastin-specific MR contrast agent (BMS-753951) was investigated for visualization and quantification vascular remodeling.^319^

#### Apoptosis

Cell apoptosis is associated with the instability of atherosclerotic plaques. In order to precisely locate and assess atherosclerotic plaque vulnerability, Li et al. conjugated targeting molecules Annexin V and radionuclide Tc-99m with thin amino-PEGs-covered-AuNPs.^320^ With the guidance of targeting molecules, SPECT/CT imaging showed an elevated accumulation of the nanoparticles in apoptotic macrophages. Intriguingly, another study revealed a promising technique for the detection of vulnerable atherosclerotic plaques by targeting apoptotic macrophages via a USPIO-based SPECT/MRI multimodal probe.^321^ Annexin A5 has been identified as a ligand to target necrotic and apoptotic cells. Therefore, Annexin A5-functionalzied micelles offer great potential for the non-invasive assessment of cell types and provided a visualization for the vulnerable atherosclerotic plaque by MRI and fluorescence imaging.^322^

Overall, the exigent demand for effective approaches for early detection and early diagnosis of vascular disorders has led to the development of several imaging techniques and contrasting agents. The challenge remains the identification of nontoxic contrast agents with longer circulation times that will allow researchers to achieve rapid and detailed imaging of tissue microstructure and lesion features. These observations open up new vistas for the clinical application of nanoparticles.

## Nanoparticle-based therapeutic methods for vascular aging-related diseases

Efforts for effective prevention of vascular aging-related disease start by encouraging people to adhere to a healthy lifestyle, such as exercising regularly, eating a healthy diet, avoiding obesity, and not smoking, among others. However, previous studies have shown that most people do not meet the requirements for healthy exercise or diet.^10^ At present, clinical management of vascular diseases chiefly includes surgical treatments and pharmacological interventions. Surgery is performed in case of acute and deteriorated situations. Notably, surgical treatments, such as endarterectomy, hematoma removal surgery, angioplasty, stenting, and coronary artery bypass grafting, are frequently conducted to ensure proper blood flow.^323^ Pharmacotherapy remains an essential approach for the treatment and prevention of vascular aging-related disorders. To this end, small molecule drugs that can regulate blood pressure, blood glucose, blood lipids, thrombus, and other pathological factors, have been developed and are currently under use. Drugs extensively used in clinical settings mainly include anti-hypertensive drugs (e.g., angiotensin-converting enzyme inhibitors), glucose-lowering drugs (e.g., metformin), lipid-lowering drugs (e.g., statins), anti-platelet drugs (e.g., clopidogrel and aspirin), anti-coagulant drugs (e.g., heparin), etc. Nevertheless, numerous pharmacological interventions have achieved limited efficacy due to poor stability, low aqueous solubility, and extensive first-pass effect. Additionally, these medications have been associated with the occurrence of severe adverse drug effects.^8^ Additionally, researchers have developed stem cell transplantation as a new attractive strategy for the treatment of vascular aging-related diseases, such as MI, ischemic stroke, and ICH. However, its clinical application has been limited by low survival rates and safety concerns.^324^ EVs have potential as a therapeutic strategy for the treatment of vascular aging-related diseases due to their excellent angiogenesis, anti-inflammation, and anti-apoptosis abilities. However, poor targeting efficiency coupled with low productivity have limited their clinical application.^325,326^

**Fig. 6:**
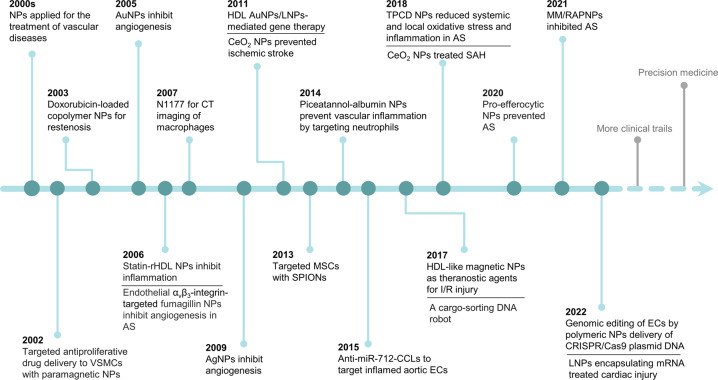
Historical timeline of nanoparticles-based therapies in vascular aging-related diseases. This timeline scheme was made using the Web of Science database. Key discoveries are highlighted. NPs nanoparticles, VSMCs vascular smooth muscle cells, AuNPs gold nanoparticles, rHDL reconstituted high-density lipoprotein, AS atherosclerosis, AgNPs silver nanoparticles, HDL high-density lipoprotein, LNPs lipid nanoparticles, CeO_2_ NPs cerium oxide nanoparticles, MSCs mesenchymal stem cells, SPIONs superparamagnetic iron oxide nanoparticles, ECs endothelial cells, SAH subarachnoid hemorrhage, I/R ischemia reperfusion, MM/RAPNPs macrophage membrane coating on the surface of rapamycin-loaded poly (lactic-co-glycolic acid) copolymer nanoparticles

Therefore, prospecting for novel efficacious therapies for the treatment of vascular aging-related disorders remains an attractive research area.^327^ Consequently, numerous studies have identified nanoparticles-based therapeutics as significant candidates for the treatment of vascular aging-related diseases^328–330^ (Fig. 6). For instance, AuNPs which mediate efficient delivery of vasoprotective, antiproliferative, and antioxidant molecules have emerged as an attractive tool for restenosis prevention, owing to its remarkable advantages over current strategies such as antiplatelet therapy and drug-eluting stents.^331^

### Nanoparticle-mediated anti-oxidative therapy

Excessive ROS accumulation and oxidative stress represent key mechanisms underlying the occurrence and development of vascular aging. Therefore, antioxidant therapy may be a promising strategy for the management of vascular aging and related diseases.^332^ Previous studies have shown that introduction of exogenous antioxidants into the biological system to scavenge excessive ROS is an effective approach to alleviate and prevent vascular diseases.^333^ Additional research evidence has shown that nano-antioxidants have the excellent antioxidant capacity and superior tolerance to harsh microenvironments in comparison to their natural counterparts.^334^ Besides, the role of mitochondrial dysfunction in vascular aging encourages the exploration of mitochondrial-targeted therapeutic modalities for the prevention and intervention of vascular aging-related diseases. Moreover, mitochondria-targeting nanoparticles for the treatment of vascular aging-related diseases have attracted considerable research attention.^335^ Nanoparticle-mediated anti-oxidative therapy has emerged as a promising strategy for the treatment of vascular aging-related diseases (Table 3).

**Table 3:** Nanoparticles-mediate anti-oxidative therapies for vascular aging-related diseases

Diseases	Nanoparticles	Therapeutic Agent	Effects	Ref(s)
Atherosclerosis	Fe_3_O_4_-CeO_2_ NPs	None	Effectively scavenge ROS	^337^
	MnO_2_ NPs	TOC	Reduce the levels of ROS and ox-LDL	^338^
	Platinum NPs	AMP	Scavenge ROS and recover compromised cell-cell junctions	^328^
	TPCD	SOD	Inhibit atherosclerosis development through eliminating excessive ROS production	^339^
	Polymeric NPs	FA	Reduce ROS production in macrophages and suppress ox-LDL up-taken	^341^
	Micelles	Simvastatin	Inhibit atherogenesis by scavenging excessive ROS, inhibiting inflammation, and decreasing cholesterol content	^582^
Hypertension	NanoSOD	None	Significantly alleviate oxidative stress through enhancing the accumulation of SOD1 protein and improving the expression of metallothionein 2	^344^
	CeO_2_ NPs	None	Ameliorate endothelium-dependent dilation and oxidative stress	^345^
	Liposomes	SOD	Reduce the blood pressure by 50 mmHg	^346^
Vascular restenosis	AuNPs	GA	Reduce the level of superoxide anion and inhibit proliferation and migration of mouse VSMCs	^330^
	Ac-bCD, Ox-bCD	Rapamycin	Serve as a pH-responsive and ROS-responsive nanoparticle and attenuate vascular restenosis	^354^
MI	PVAX	None	Significantly attenuate ROS production by decreasing the expression of NOX2 and NOX4	^356^
	PEGylated liposomes	NM-aFGF	Improve the myocardial structural by inhibiting myocardial oxidative stress	^359^
	Fullerene	None	Regulate Nrf2/ARE-antioxidant signaling pathway	^357^
	Polymeric NPs	CoQ10	Substantially improve ejection fraction	^358^
Ischemic stroke	CeO_2_ NPs	None	Inhibit ischemic stroke development by suppressing apoptosis and scavenging excessive ROS	^368^
	CeO_2_ NPs	None	Effectively cross BBB and access brain tissues via a receptor-mediated transcytosis pathway	^370^
	PEGylated CeO_2_ NPs	None	Protect against ROS-induced cell death	^368^
	CeO_2_@ZIF-8 NPs	None	Significantly reduce oxidative stress-induced apoptosis and tissue injury	^329^
	Platinum NPs	None	Pronouncedly inhibit the production of superoxide anion and reduce oxidative stress-induced MMP-9 activation	^372^
	PEG-modified Fe_3_O_4_ NPs	None	Promote BBB reconstruction	^369^
ICH	CeO_2_ NPs	None	Effective scavenge ROS and inhibit NF-κB signal pathway	^375^
	CeO_2_ NPs	None	Significantly reduce neuronal death, and macrophage infiltration by enhancing antioxidative effect	^376^
	PEG-CeO_2_ NPs	None	Suppress ROS-related NF-κB activation	^377^
	t-PA@iRNPs	None	Inhibit subarachnoid hemorrhage via the elimination of excessive ROS	^378^
	PLGA NPs	Curcumin	Remarkably suppress subarachnoid hemorrhage-induced oxidative stress	^379^
Vascular dementia	SLNs	Resveratrol	Reduce the production of ROS and lipid peroxidation	^380^
CKD	C-Mn_3_O_4_ NPs	None	Alleviate intracellular ROS production and maintain cellular redox balance	^383^

*NPs* nanoparticles, *ROS* reactive oxygen species, *TOC* D-α-tocopherol, *ox-LDL* oxidized low-density lipoprotein, *AMP* 2-amino-6-mercaptopurine, *SOD* superoxide dismutase, *FA* ferulic acid, *NanoSOD* copper/zinc SOD nanoformulation, *AuNPs* gold nanoparticles, *GA* ginkgolide A, *VSMCs* vascular smooth muscle cells, *Ac-bCD* acetalated β-cyclodextrin material, *Ox-bCD* β-cyclodextrin material, *MI* myocardial infarction, *NOX2* NADPH oxidase 2, *NM-aFGF* non-mitogenic acidic fibroblast growth factor, *Nrf2* nuclear factor erythroid 2-related factor 2, *ARE* antioxidant response element, *CoQ10* Coenzyme Q10, *BBB* blood-brain barrier, MMP-9 matrix metalloproteinase-9, NF-κB nuclear factor-kappaB, ICH Intracerebral hemorrhage, PEG poly(ethylene glycol), t-PA@iRNPs tissue plasminogen activator-installed, nitroxide radical-containing, self-assembled polyion complex nanoparticles, *PLGA* poly lactic-co-glycolic acid, *SLNs* solid lipid nanoparticles, *CKD* chronic kidney disease

#### Vascular aging-related cardiovascular diseases

Atherosclerosis is strongly associated with multiple vascular disorders, such as ischemic stroke, ischemic heart disease, and peripheral arterial disease.^336^ Given the crucial effect of oxidative stress in atherogenesis, antioxidant therapy has emerged as a promising strategy for the prevention of atherosclerosis.^150^ However, the currently available antioxidants have exhibited limited efficacy in managing the condition.^9^ Accumulating evidence suggested that nanoparticle-based therapeutic modalities, targeting or scavenging excessive ROS, are potential anti-atherosclerotic therapies. Notably, nanoenzymes, such as CeO_2_ and MnO_2_ nanoparticles, have shown promise for the treatment of atherosclerosis due to their excellent biocompatibility, high stability, and anti-oxidative properties. Additionally, novel Fe_3_O_4_-CeO_2_ core-shell nanoparticles were found to be promising platforms for the diagnosis and treatment of ROS-related vascular disorders due to their excellent MRI ability and ROS scavenging performance.^337^ Bizeau et al. constructed hyaluronic acid (HA)-coated spherical MnO_2_ microparticles for controlling drug release and scavenging excessive ROS.^338^ Moreover, platinum nanoparticles were also shown to serve as ROS scavengers and play a role in reversing cell junctions damage under hyperlipidemic and hyperglycemic conditions.^328^ In another study, Wang et al. generated a broad-spectrum ROS-eliminating material called TPCD nanoparticles.^339^ After intravenous injection, TPCD nanoparticles predominantly localized in atherosclerotic plaques in vivo and markedly suppressed atherosclerosis progression. Mechanistically, TPCD nanoparticles can be efficaciously and promptly internalized by both VSMCs and macrophages. Notably, TPCD nanoparticles not only alleviated macrophage inflammation and cell apoptosis by eliminating excessive intracellular ROS production, but also repressed the formation of foam cells by attenuating the internalization of ox-LDL.^339^ Moreover, researchers have encapsulated several therapeutic agents in nanoparticles with the aim of enhancing their abilities to decrease LDL uptake and ROS production. Moreover, nanoformulations synthesized by loading D-α-tocopherol (TOC) with MnO_2_ microparticles were found to effectively suppress levels of ROS and LDL oxidation.^338^ Ferulic acid (FA), a free radical scavenger, has been approved as a food additive for the prevention of lipid peroxidation.^340^ Additionally, FA-based poly(anhydride-ester) nanoparticles can overcome the deficiencies of FA in dose, stability, and targeted delivery, thus have potential as a valuable platform for the management of atherosclerosis.^341^

Prevalence of hypertension is on the rise, owing to an increase in the aging population.^342^ Notably, oxidative stress promotes hypertension progression through regulation of vascular functions, inflammation, and aldosterone/mineralocorticoid actions. Previous studies have shown that increased activation and upregulation of NOXs in hypertension are critical mechanisms underlying the occurrence of oxidative stress in vascular aging-related cardiovascular disease.^158,343^ For example, copper/zinc SOD nanoformulation was shown to significantly mediate a decrease in the level of oxidative stress by increasing the accumulation of SOD1 protein and improving the expression of metallothionein 2 in ECs.^344^ Another study showed that intravenous injection of CeO_2_ nanoparticles ameliorated endothelium-dependent dilation and oxidative stress in spontaneously hypertensive rats (SHRs) relative to saline alone.^345^ Besides, SOD-loaded liposomes mediated a decrease in blood pressure by 50 mmHg in angiotensin II-induced hypertension rat models.^346^

Heart failure is a severe public health problem worldwide. A previous study demonstrated that inhalation-based delivery of TPCD nanoparticles suppressed doxorubicin-induced heart failure in mice due to internalization in cardiomyocytes and scavenging excessive ROS.^347^ Moreover, Vanillyl alcohol (PVAX)-polymer nanoparticles treatment alleviated doxorubicin-induced cardiomyopathy by inhibiting activation of poly (ADP ribose) polymerase 1 (PARP-1) and caspase-3.^348^ Experimental results from a rodent diabetic cardiomyopathy model revealed that inhalation of calcium phosphate nanoparticles loaded with a therapeutic mimetic peptide markedly improved myocardial contraction and cardiac function via rapid translocation of calcium phosphate nanoparticles from pulmonary to myocardium, where the therapeutic mimetic peptide is quickly released.^349^

Vascular restenosis is associated with proliferation and migration of VSMCs, as well as synthesis and remodeling of the ECM.^350^ ROS is a critical regulator for enhancing VSMCs proliferation and migration.^351^ Emerging studies have indicated that antioxidant therapies have potential efficacy against vascular restenosis after angioplasty.^352,353^ Moreover, Acetalated β-cyclodextrin material (Ac-bCD) serves as a PH-responsive drug carrier, whereas hydrophobic functionalization of β-cyclodextrin material (Ox-bCD) functions as a ROS-responsive drug delivery system. Experimental results revealed that intravenous administration of pH-responsive or ROS-responsive nanoparticles effectively alleviated neointimal hyperplasia in comparison to non-responsive PLGA nanoparticles-based therapy.^354^

MI is the most harmful type of ischemic heart diseases, resulting in loss of tissue and impaired heart function.^355^ Notably, overproduction of ROS represents the primary cause of myocardial I/R-mediated tissue damage. Previous studies have reported the application of nanoparticles for scavenging excessive ROS in MI. For instance, Bae et al. prepared hydrogen peroxide (H_2_O_2_)-responsive antioxidant polymeric nanoparticles and named them HPOX and PVAX.^356^ The authors found that a single injection of PVAX remarkably ameliorated fraction shortening and cardiac output, reduced infarction size, and downregulated NOX2 and NOX4 expression compared to PLGA nanoparticles. Besides, PVAX also effectively inhibited the activation of caspase-3, reduced the number of TUNEL-positive cells, and downregulated the levels of tumor necrosis factor alpha (TNF-α) and MCP-1 mRNA.^356^ Under oxidative stress conditions, C(60) fullerene enhanced antioxidant capacity of rat heart tissue and attenuated lipid peroxidation by inhibiting ROS production and suppressing the release of O_2_·^−^ and H_2_O_2._^357^ Coenzyme Q10 (CoQ10) plays a critical role in the mitochondrial electron transport chain. Polymeric nanoparticles encapsulated CoQ10 for the management of MI, with oral administration of CoQ10-loaded nanoparticles found to substantially improve ejection fraction in female Sprague-Dawley rats with myocardial ischemia.^358^ Additionally, PEGylated liposomes encapsulated non-mitogenic acidic fibroblast growth factor (aFGF) has the ability to protect against diabetic cardiomyopathy-induced oxidative stress by activating the Akt/glycogen synthase kinase (GSK)/nuclear factor erythroid 2-related factor 2 (Nrf2) signaling pathway.^359^ Additional studies have shown that Cyclosporin A is a therapeutic drug for the treatment of myocardial I/R injury by suppressing the opening of mitochondrial permeability transition pore (mPTP).^360^ However, the clinical application of cyclosporin A is limited by its immunosuppressive effect on other normal organs and tissues. SS31 is a novel mitochondrial targeting peptide that can guide drug accumulation in the mitochondria.^361^ Experimental results revealed that cyclosporin A-loaded PLGA-PEG-SS31 conferred excellent cardioprotective effects against MI/RI in rat hearts by directly delivering cyclosporin A to the mitochondria and protecting mitochondrial integrity.^362^

Several natural polyphenols, such as resveratrol, quercetin, and curcumin, play a role in suppressing ROS production. Notably, nanoparticles have emerged as promising vehicles for the delivery of polyphenols to targeted tissues.^363^ A previous study demonstrated that resveratrol-SLNs showed stable under storage and sustained release profile. Moreover, resveratrol-SLNs exerted a therapeutic effect on doxorubicin-induced cardiotoxicity in mice,^364^ while resveratrol-loaded liposomes promoted mitochondrial respiratory capacity in myocardial cells.^365^ On the other hand, Quercetin-MSNs promoted the cardioprotective effects on myocardial I/R injury rats by significantly enhancing the activity of the Janus kinase 2 (JAK2)/signal transducer and activator of transcription 3 (STAT3) signaling pathway.^366^ Additional studies have shown that curcumin nanoparticles can protect against doxorubicin-induced cardiotoxicity by inhibiting doxorubicin-induced significant increase in lipid peroxidation (MDA), NO, acetycholinesterase (AchE), and lactate dehydrogenase (LDH), as well as modulating a doxorubicin-induced decrease in glutathione (GSH), norepinephrine (NE) and serotonin (5-HT), and ATPase.^367^

#### Vascular aging-related cerebrovascular diseases

Ischemic stroke is a severe vascular aging-related cerebrovascular disease that causes disability and death. Previous studies have implicated oxidative stress in the activation of apoptosis, necrosis, and autophagy pathways, as well as induction of cerebral vasculature damage, ischemic injury, and disruption of the blood-brain barrier (BBB).^160^ Additionally, studies have revealed that metallic nanoparticles, such as CeO_2_, platinum, and Fe_3_O_4_ nanoparticles, serve as ROS scavengers. For example, Kim et al. found that CeO_2_ nanoparticles with a size of 3 nm could effectively prevent ischemic stroke by suppressing apoptosis and scavenging excessive ROS.^368^ In addition, PEGylated CeO_2_ nanoparticles exerted a significant protective effect against ROS-induced cell death, whereas PEG-modified Fe_3_O_4_ nanoparticles beneficial for BBB reconstruction.^368,369^ However, the accumulation of therapeutic nanoparticles at the brain injury site is limited by BBB’s integrity.

The development of therapeutic nanoparticles that can cross the BBB has attracted numerous research attention. For instance, Bao et al. prepared PEG and Angiopep-2-modified CeO_2_ nanoparticles and found that they effectively crossed BBB and accessed brain tissues via a receptor-mediated transcytosis pathway.^370^ Another study found that CeO_2_ nanoparticles coated with zeolitic imidazolate framework-8 (CeO_2_@ZIF-8 NPs) exhibited the enhancement of BBB penetration ability, the extension of blood circulation, and the reduction of clearance rate. Results from an in vivo study demonstrated that CeO_2_@ZIF-8 NPs administration significantly suppressed oxidative stress-induced apoptosis and tissue injury in middle cerebral artery occlusion mice.^329^ Additionally, platinum nanoparticles exhibited excellent neuroprotective effects against ischemic stroke, with their administration markedly inhibiting the production of superoxide anion and reducing oxidative stress-induced MMP-9 activation in transient middle cerebral artery occlusion mice.^371,372^

ICH, a disorder characterized by high morbidity and mortality, currently has no effective treatment therapies.^373,374^ Previous studies have shown that CeO_2_ nanoparticles play a role in altering microglial from pro-inflammatory M1 to anti-inflammatory M2 phenotype, through effective scavenging for ROS and inhibition of the NF-κB signal pathway.^375^ Experimental results revealed that intravenous injection of CeO_2_ nanoparticles exhibited potent anti-oxidative, cytoprotective, and anti-inflammatory activities in vitro and remarkably alleviated neuronal death, macrophage infiltration, and brain edema in vivo.^376^ Treatment of collagenase VII-induced intracerebral hemorrhage mice with PEG-CeO_2_ nanoparticles resulted in marked inhibition of ROS-related NF-κB activation and suppression of expression of A1 astrocytes and M1 microglia, ultimately promoting remyelination.^377^ Besides, tissue plasminogen activator (t-PA)-installed, nitroxide radical-containing, self-assembled polyion complex nanoparticles (t-PA@iRNPs) suppressed t-PA-induced subarachnoid hemorrhage by eliminating excessive ROS production.^378^ On the other hand, curcumin-PLGA nanoparticles remarkably inhibited subarachnoid hemorrhage-induced oxidative stress and ameliorated neurological function compared to curcumin.^379^

Vascular dementia, the leading cause of cognitive decline resulting from vascular lesions, causes about 15% of all dementia cases.^203,380^ It has been reported that oxidative stress and mitochondrial dysfunction play a role in cognitive decline. Yadav et al. demonstrated that resveratrol-loaded SLNs were highly protective against vascular dementia.^380^ In addition, resveratrol-loaded SLNs treatment resulted in a strong reduction of ROS production, lipid peroxidation, and protein carbonyls as well as potent enhancement of redox ratio and MnSOD activity. Besides, the level of hypoxia-inducible factor 1α (HIF-1α) was decreased, whereas the expression of Nrf2 and heme oxygenase 1 (HO-1) were increased.^380^

#### Vascular aging-related chronic kidney disease

Chronic kidney disease is defined as a glomerular filtration rate of less than 60 ml/min per 1.73 m^2^ or a urinary albumin-to-creatinine ratio exceed 30 mg/g.^381^ The prevalence of chronic kidney disease is on the rise, owing to an increase in the aging population coupled with the rapid increase in obesity, diabetes, and hypertension.^382^ At present, only a handful of therapies exist for the treatment of chronic kidney disease. In fact, these therapies can only delay disease progression, a phenomenon that necessitates urgent development of new effective therapeutic modalities to either stop or reverse disease progression. Nanoparticles have been implicated in the intervention and prevention of chronic kidney disease, while ROS imbalance and associated mitochondrial dysfunction have been strongly associated with the development and progression of chronic kidney disease. For example, citrate-functionalized Mn_3_O_4_ nanoparticles (C-Mn_3_O_4_ NPs) were found to play a role in reducing intracellular ROS and maintaining cellular redox balance in the oxidative injury-mice model. Notably, four weeks of C-Mn_3_O_4_ NPs treatment effectively restored renal function, mediated recovery of kidney architecture, improved expression of pro-inflammatory factors, and suppressed glomerulosclerosis and interstitial fibrosis in cisplatin-induced chronic kidney disease mice model.^383^

### Nanoparticle-mediated anti-inflammatory therapy

Vascular inflammation is strongly associated with vascular aging and related disorders, thus immune-modulatory strategies have potential as therapeutic modalities for the treatment of inflammation-related vascular diseases.^384,385^ Nevertheless, the application of many anti-inflammatory drugs is largely limited by pharmacokinetics and route of administration, such as short half-life, low stability, low bioavailability, and occurrence of side effects. Previous studies have shown that nanoparticles loaded with anti-inflammatory drugs, such as rapamycin, methotrexate, celecoxib, curcumin, colchicine, resveratrol, and wogonin, conferred effective protection against vascular diseases by suppressing inflammatory responses.^386–392^ In addition, several lipid- and glucose-lowering drugs, such as statins, pioglitazone, rosiglitazone, liraglutide, and exenatide, exerted beneficial effects on cardiovascular disorders.^393–395^ On the basis of traditional medicine, targeted anti-inflammatory therapy has emerged as a promising approach for reducing residual cardiovascular risk.^396^ Numerous studies have revealed that nanoparticles are ideal platforms for the delivery of anti-inflammatory reagents, which can improve the anti-inflammatory effects of drugs. Nanoparticle-mediated anti-inflammatory therapy has been implicated in the treatment of vascular aging-related diseases (Table 4).

**Table 4:** Nanoparticles-mediated anti-inflammatory therapies for vascular aging-related diseases

Diseases	Nanoparticles	Therapeutic Agent	Effects	Ref(s)
Atherosclerosis	PFN1-CD-IONs	Rapamycin	Inhibit atherosclerosis progression	^397^
	Liposomes	Methotrexate	Reduce the expression of IL-1β, IL-6, and TNF-α	^401^
	LDEs	Methotrexate, paclitaxel	Increase the anti-atherosclerosis effects through strongly reducing the number of macrophages and the expression of MMP-9 and TNF-α	^402^
	LDEs	Docetaxel	Dramatically alleviate the production of pro-inflammatory cytokines, such as IL-1β, IL-6, and TNF-α	^403^
	LDEs	Carmustine	Reduce pro-inflammatory molecules expression	^404^
	LDEs	Methotrexate	Decrease the generation of pro-inflammatory factors, including IL-1β, IL-18, TNF-α, MCP-1, MMP-9, MMP-12 and increase anti-inflammatory IL-10 expression	^583^
	rHDL NPs	TRAF-STOP	Effectively inhibit macrophages migration and activation through the downregulation of intermediates phosphorylation of the canonical NF-κB pathway	^405^
	Ac-bCD	Rapamycin	Remarkably enhance plaques stability and reduce atherosclerotic lesions	^398^
	Polymeric NPs	Pioglitazone	Inhibit MMPs and cathepsins activation	^399^
	SPNs	Methotrexate	Dramatically inhibit pro-inflammatory molecules production, including IL-6 and TNF-α	^400^
Hypertension	CeO_2_ NPs	None	Enhance the expression of IL-10 and TNF-α	^345^
Vascular restenosis	CuBiS_2_ NPs	None	Suppress inflammation through eliminating macrophages	^410^
	Liposomes	Alendronate	Attenuate restenosis by eliminating circulating monocytes/macrophages	^584^
	Polypyrrole NPs	None	Remarkably suppress vascular inflammation and stenosis through eliminating infiltrating macrophages	^411^
MI	ApoA-I NPs	None	Attenuate myocardial infarction by decreasing the systemic and cardiac inflammatory response	^421^
	AuNPs	None	Ameliorate cardiac systolic function by alleviating the accumulation of TNF-α	^414,415,417^
	Liposomes	Rapamycin	Inhibit macrophages polarization and attenuate excessive inflammation following MI	^418^
	LDEs	Methotrexate	Improve left ventricular systolic function through enhancing antioxidant enzymes and reducing the number of inflammatory cells	^390^
	NPs	Curcumin	Inhibit the expression of inflammatory cytokines, such as IL-1α, IL-1β, IL-6, TNF-α, MCP-1, and RANTES	^419,420,585^
	PLGA NPs	Pitavastatin	Significantly reduce the accumulation of monocytes/macrophages	^393^
	PLGA NPs	pioglitazone	Protect against cardiac remodeling by suppressing monocyte-mediated acute inflammation	^394^
	PLGA NPs	Celecoxib	Hamper the development of heart failure	^391^
Ischemic stroke	Selenium NPs	OX26	Inhibit excessive inflammation and oxidative metabolism	^423^
	CeO_2_@ZIF-8 NPs	None	Induce suppression of astrocytes activation and pro-inflammatory factors secretion	^329^
	MnO_2_ NPs	Fingolimod	Inhibit ischemic stroke by reducing oxidative stress and modulating inflammatory microenvironment	^424^
	PEG NPs	Melanin	Reduce oxidative stress and inflammatory factors production	^425^
	NLCs	Resveratrol	Ameliorate oxidative stress and reduce the activation of IL-1β, IL-1, and TNF-α in ischemic stroke animal models	^105^
	NPs	Rapamycin	Inhibit the proliferation of inflammatory cells	^426^
	Membrane-derived nanovesicle	Resolvins	Significantly enhance therapeutic efficacy in treating ischemic stroke	^427^
	PEG NPs	Tanshinone IIA	Possess remarkable neuroprotective effects on ischemic stroke by regulating inflammatory cascades and neuronal signal pathways	^429^
ICH	PLGA NPs	Curcumin	Significantly inhibit inflammatory responses and microglia activation in subarachnoid hemorrhage-induced BBB disruption	^379^
Vascular dementia	Liposomes	GM1	Reverse medin-induced ECs immune activation	^432^
Chronic kidney disease	AuNPs	Artificial kidney	Reduce inflammatory responses	^433^
	LNPs	Rapamycin	Effectively inhibit podocytes-induced inflammatory responses	^106^
	PLGA NPs	Resveratrol	Potential be a promising approach for preventing chronic kidney disease by reducing the secretion of NLRP3 inflammasome and IL-1β	^434^
	PLGA NPs	EB	Protect against renal fibrosis via Smad3-dependent mechanism	^437^

*PFN1* profilin-1 antibody, *IONs* iron oxide nanoparticles, *LDEs* lipid core nanoparticles, *IL-1β* interleukin-1β, *TNF-α* tumor necrosis factor alpha, *MMP-9* matrix metalloproteinase-9, *MCP-1* monocyte chemotactic protein, *rHDL* recombinant high-density lipoprotein, *NPs* nanoparticles, *NF-κB* nuclear factor-kappaB, *Ac-bCD* acetalated β-cyclodextrin material, *SPNs* spherical polymeric nano-constructs, *AuNPs* gold nanoparticles, *MI* myocardial infarction, *PLGA* poly lactic-co-glycolic acid, *OX26* anti-transferrin receptor monoclonal antibody, *PEG* poly(ethylene glycol), *NLCs* nanostructured lipid carriers, *ICH* Intracerebral hemorrhage, *LNPs* lipid nanoparticles, *BBB* blood-brain barrier, *ECs* endothelial cells, *GM1* monosialoganglioside, *EB* Eleutheroside B, *NLRP3* NOD-like receptor family pyrin domain containing 3

#### Vascular aging-related cardiovascular diseases

Atherosclerosis has been recognized as a low-grade chronic inflammatory disease. Nanoparticles combined with anti-inflammatory compounds may be an effective approach to target pro-inflammatory mediators within atherosclerotic plaques, thus aid in regulating inflammation and vascular cell function.^131^ Profilin-1 antibody-functionalized IONs served not only as multifunctional imaging probes but also as carriers for the delivery of rapamycin.^397^ Subcutaneous injection of rapamycin-acetalated β-cyclodextrin remarkably increased plaques stability and significantly suppressed the formation of atherosclerotic lesions by selectively repressing the mechanistic target of rapamycin complex 1 (mTORC1), whereas its oral administration simultaneously suppressed both mTORC1 and mTORC2. Additional evidence revealed a significant reduction in rupture-prone pro-inflammatory factors in serum and aorta following treatment.^398^ Moreover, pioglitazone loaded into PLGA nanoparticles regulate the expression of inflammatory cytokines and inhibits the activation of MMPs and cathepsins.^399^ Spherical polymeric nano-constructs (SPNs) enveloping methotrexate were accumulated in atherosclerotic plaques and engulfed by macrophages. Next, methotrexate-SPNs released their anti-inflammatory substances in macrophages, thereby dramatically inhibiting the production of pro-inflammatory molecules, including IL-6 and TNF-α.^400^ Besides, liposomes-mediated methotrexate delivery mediated upregulation of ATP binding cassette transporter A1 (ABCA1) and exhibited a significant anti-inflammatory effect by downregulating the expression of IL-1β, IL-6, and TNF-α.^401^ In another study, Gomes et al. found that combining methotrexate-lipid core nanoparticles (LDEs) with paclitaxel-LDEs could effectively enhance the anti-atherosclerosis effects by strongly reducing the number of macrophages, the area of atherosclerotic lesions, and downregulating the expression of MMP-9 and TNF-α.^402^

Docetaxel carried in LDE dramatically alleviated vascular inflammation by downregulating the expression of TGF-β, MMP-2, MMP-9, collagen 1 and 3 and mitigating the production of pro-inflammatory cytokines, including NF-κB, IL-1β, IL-6, TNF-α, and von Willebrand factor. Besides, the number of macrophages also decreased after docetaxel-LDE treatment. Further evidence indicated that intravenous injection of docetaxel-LDE resulted in an 80% reduction of atheroma area compared to LDE administration alone. Notably, docetaxel-LDE treatment was not associated with any hematological, renal, or hepatic toxicity in rabbit models.^403^ Additionally, carmustine loaded into LDE mediated downregulation of pro-inflammatory molecules, the number of VSMCs and macrophages, and the area of the atherosclerotic lesions.^404^ On the other hand, TRAF-STOP carried in rHDL nanoparticles overcame immune suppression of long-term CD40 treatment in atherosclerosis, and effectively attenuated migration and activation of macrophages by downregulating intermediates phosphorylation of the canonical signaling NF-κB pathway.^405^ The development of ROS-responsive anti-inflammatory nanoparticles can be applied for targeted treatment of oxidative stress- and inflammation-related disorders.^406^ Additionally, Sun et al. formed ROS-responsive nanoplatforms for drug delivery via covalently self-assembled polymer nanocapsules. ROS-responsive payload release from luminol-loaded polymer nanocapsules reportedly exhibited excellent anti-inflammatory effects both in vitro and in vivo.^407^ Additional evidence has shown that macrophage membrane-coated rapamycin-loaded PLGA nanoparticles delay atherosclerosis progression by effectively suppressing phagocytosis by macrophages and targeted activated ECs.^408^

Hypertension and vascular restenosis are closely related to vascular inflammation.^409^ To date, however, only a handful of studies have evaluated the potential for nanoparticles for the delivery of anti-inflammatory drugs for hypertension and vascular restenosis management. Minarchick et al. found that injection of CeO_2_ nanoparticles regulated inflammation by upregulating IL-10 and TNF-α expression in Wistar-Kyoto rats (WKYs) and suppressing leukocyte flux in SHRs.^345^ Additionally, Wu et al. developed a novel multifunctional CuBiS_2_ nanoparticle for CT imaging-guided photothermal therapy for the prevention of artery restenosis, and found that these nanoparticles inhibited inflammation by eliminating macrophages.^410^ Local injection of polypyrrole nanoparticles, combined with 915 nm near-infrared laser irradiation, remarkably attenuated both vascular inflammation and stenosis through eliminating infiltrating macrophages.^411^

In infarcted hearts, necrotic cells trigger myocardial and systemic inflammatory responses. Excessive, long-term, and dysregulated inflammation contributes to heart failure following infarction.^412^ Notably, AuNPs have emerged as ideal drug delivery systems for the intervention and prevention of cardiovascular diseases, due to their cardioprotective effects and unique properties, such as safety and prolonged drug action.^413–415^ For instance, the accumulation of AuNPs in infarcted heart tissues reportedly decreased the size of infarction, suppressed levels of TNF-α and cardiac fibrosis, and ameliorated cardiac systolic function.^416,417^ MI antigens and rapamycin-loaded liposomes induced antigen-specific regulatory T cells and suppressed macrophage polarization, thereby blocking excessive inflammation following MI.^418^ Methotrexate carried in LDEs improved left ventricular systolic function, by enhancing antioxidant enzymes and suppressing the number of inflammatory cells. Additionally, Methotrexate-LDEs also alleviated infarction size, myocyte hypertrophy and necrosis, and myocardial fibrosis in left coronary artery ligation-treated Wistar rats.^390^ Margulis et al. demonstrated that celecoxib-nanoparticles effectively antagonized heart failure post-MI by promoting angiogenesis of ischemic myocardium.^391^ Experimental results, from isoproterenol-induced rat MI models, revealed that the gavage of curcumin nanoparticles effectively improved oxidative stress and inhibited the expression of inflammatory cytokines, such as IL-1α, IL-1β, IL-6, TNF-α, MCP-1, and RANTES, compared to conventional curcumin. Additionally, the authors noted a marked reduction in the levels of MMP-2 and MMP-9. Histopathological results further demonstrated that curcumin nanoparticles efficiently prevented myocardial necrosis and attenuated neutrophil infiltration and interstitial edema compared to curcumin.^419^ Another study also showed that curcumin nanoparticles exhibited a protective effect on isoproterenol-induced MI by suppressing oxidative stress, electrocardiogram, and biological changes in the myocardial tissue.^420^ Besides, pitavastatin-loaded nanoparticles significantly attenuated the accumulation of monocytes/macrophages and suppressed cardiac post-infarct remodeling.^393^ Experimental results from mouse MI models revealed that polymeric nanoparticles containing pioglitazone targeted inflammatory monocytes thereby protecting the heart from cardiac remodeling through suppressing monocyte-mediated acute inflammation and improving cardiac healing.^394^ Moreover, a single intravenous injection of ApoA-I nanoparticles after reperfusion instantly mitigated the systemic and cardiac inflammatory responses in a preclinical MI mouse model. Mechanistically, the administration of ApoA-I nanoparticles significantly reduced the number of circulating leukocytes and leukocytes recruited to the ischemic heart, mainly due to the reduction of plasma cardiac troponin-I. Besides, ApoA-I nanoparticles reduced the recruitment of neutrophils and monocytes to the ischemic heart by suppressing the cardiac expression of chemokines. Another study found that ApoA-I nanoparticles were preferentially bound to pro-inflammatory monocytes via scavenger receptor BI (SR-BI).^421^

#### Vascular aging-related cerebrovascular diseases

Post-stroke immune responses are novel breakthrough targets for treating ischemic stroke.^422^ Amani et al. showed that selenium nanoparticles exerted a therapeutic effect on ischemic stroke by regulating inflammatory and metabolic signaling pathways, such as the JAK2/STAT3 and mTOR-related signaling pathways.^423^ Additionally, CeO_2_@ZIF-8 NPs were efficacious in treating stroke by inhibiting astrocyte activation and pro-inflammatory factors secretion.^329^ Researchers have also combined several anti-inflammatory agents, such as melanin, resveratrol, rapamycin, and curcumin, with nanoparticles to improve their efficacy and bioavailability. Fingolimod-macrophage-disguised honeycomb MnO_2_ nanoparticles reversed the brain pro-inflammatory microenvironment through consuming excessive H_2_O_2_ and promoting M1 microglia switch to M2 phenotype.^424^ Results from an ischemic stroke rat model and in vitro studies revealed that bioinspired melanin nanoparticles had excellent antioxidant effects. Apart from reducing oxidative stress, melanin nanoparticles reportedly play a role in alleviating the production of inflammatory factors.^425^ Particularly, resveratrol-loaded nanoparticles ameliorated oxidative stress and reduced the activation of IL-1β, IL-1, and TNF-α in ischemic stroke animal models.^105^ Monocyte membrane-coated rapamycin nanoparticles (McM/RNPs) can be applied for stroke treatment, owing to their efficacy in suppressing microglia proliferation and blocking monocyte infiltration. Besides, McM/RNPs can actively target and bind to inflammatory ECs, thus can serve as a shield between monocytes and ECs.^426^ It has been revealed that resolvin D2 exhibited a critical role in the modulation of inflammation and tissue repair. Membrane-derived nanovesicles-encapsulated resolvin D2 pronouncedly enhanced its therapeutic efficacy in treating murine ischemic stroke.^427^ In acerebral I/R injury in stroke animal model, curcumin-loaded triblock copolymer nanomicelles effectively downregulated the expression of NF-κB-p65 protein and inflammatory cytokines, including IL-1β, IL-6, and TNF-α.^428^ In addition, cationic bovine serum albumin-conjugated tanshinone IIA PEGylated nanoparticles exhibited a conspicuous neuroprotective effect on ischemic stroke by participating in the regulation of inflammatory and neuronal signaling pathways.^429^

Growing evidence has revealed that inflammation plays an important role in ICH and vascular dementia development.^430,431^ Curcumin-PLGA nanoparticles significantly inhibited inflammatory responses and microglia activation relative to curcumin alone. Besides, protection of tight junction proteins, including occludin, claudin-5, and ZO-1 by curcumin-PLGA nanoparticles reportedly alleviated BBB dysfunction after subarachnoid hemorrhage.^379^ Additionally, patients with vascular dementia exhibited higher medin in their cerebral artery compared to their cognitively normal counterparts. Notably, medin is involved in ECs immune activation and astrocyte activation, which can be reversed by liposomes-encapsulated monosialoganglioside.^432^

#### Vascular aging-related chronic kidney disease

Chronic kidney disease is an inflammation-associated disorder. Chen et al. prepared a resonantly illuminated AuNPs-modified artificial kidney (AuNPs@AK) for treating chronic kidney disease. This therapy not only achieved anti-inflammatory, anti-thrombotic, and anti-oxidative effects in patients with chronic kidney disease complicated with hemodialysis, but was also accompanied by multiple advantages, evidenced by shorter treatment times and low risk of adverse reactions.^433^ Other studies have shown that resveratrol-loaded nanoparticles have the potential to prevent chronic kidney disease through the suppression of secretion of NOD-like receptor family pyrin domain containing 3 (NLRP3) inflammasome and IL-1β.^434^ VCAM-1, a surface-expressed receptor, plays a major role in promoting receptor-mediated endocytosis of nanoparticles-based drugs. VCAM-1-decorated lipid-based nanocarriers loaded with rapamycin effectively suppressed podocytes-induced inflammatory responses.^106^ Additionally, intravenous infusion of SPIONs has been applied to diagnose and treat iron deficiency anemia in adults with chronic renal failure.^435^ Hemodialysis is crucial for kidney diseases. Notably, plasmon-induced dialysate comprising AuNPs reduced the time required for elimination of 70% creatinine and blood urine nitrogen by 59% and 47%, respectively, compared to conventional deionized water. Concurrently, NO release from lipopolysaccharide-treated inflammatory cells was inhibited.^436^ Although renal fibrosis is a common complication of chronic kidney disease, no effective treatment for this condition has exists at present. Researchers have employed PLGA nanoparticles for eleutheroside B delivery and enhanced eleutheroside B bioavailability, with small animal imaging revealing that eleutheroside B-PLGA nanoparticles can selectively accumulate in mice kidneys for up to 7 days.^437^

### Nanoparticle-mediated anti- and pro- proliferation and anti-apoptotic therapy

Endothelial dysfunction and VSMCs proliferation are major contributors to vascular aging and are strongly correlated with diverse vascular aging-related diseases.^438^ Numerous studies have shown that nanoparticles can be exploited to target and regulate vascular endothelial and VSMCs functions, including cell proliferation, migration, inflammation, senescence, and apoptosis.^36,339,439–441^ There are particularly strong data indicating that proliferation and migration of endothelial dysfunction and VSMCs are vital to vascular aging-related diseases, such as atherosclerosis, hypertension, vascular stenosis and restenosis, and MI.^175,176^ Therefore, nanoparticle-mediated anti-proliferation and anti-apoptotic therapies hold great promise in preventing vascular aging-related disorders.

Nanoparticle-targeted cellular lifecycle provides novel insights to guide the development of therapies for atherosclerosis. Previous studies have demonstrated that physically synthesized AuNPs (pAuNPs) play a critical role in regulating the proliferation and migration of VSMCs in balloon-injured rat carotid arteries. Mechanistically, naked pAuNPs exert an inhibitory effect on focal adhesion kinase (FAK) phosphorylation and collagen-induced tyrosine-protein activation. Additionally, they also suppressed platelet-derived growth factor (PDGF)-induced VSMCs proliferation and migration in vivo.^440^ Additional evidence showed that naked pAuNPs stimulated a redox-related reaction and promote p38 mitogen-activated protein kinase (MAPK) activation, thereby inducing activation of Nrf2. Notably, the elevated HO-1 levels in VSMCs were mediated by naked pAuNPs-inducing Nrf2 phosphorylation, expression, and translocation into the nucleus.^442^ Another study showed that novel nanoparticles-cationic lipid microbubble complex-mediated aFGF, combined with ultrasound targeted microbubble destruction, inhibited doxorubicin-induced heart failure by attenuating apoptosis and promoting angiogenesis.^443^ H_2_O_2_-responsive MSNs, loaded with captopril, were highly efficacious in zebrafish with KillerRed-induced heart failure.^444^

Numerous nanoparticle-carried anti-proliferation drugs, such as heparin, polyphenolic, liver X receptor (LXR) agonist, docetaxel, and paclitaxel, have shown increased therapeutic efficiency. For example, low doses of heparin-coated IONs significantly increased the proliferation of ECs and inhibited that of VSMCs.^445^ Additionally, polyphenolic and AuNPs-conjugated graphene nanosheets (Polyp-Au-GO) inhibited proliferation and growth of VSMCs through blocking the G1 cell cycle, downregulating cyclin, downregulating extracellular signal-regulated kinase 1/2 (ERK1/2) phosphorylation, and alleviating TNF-R-evoked inflammatory responses. Besides, Polyp-Au-GO also suppressed coronary ECs proliferation.^446^ Previous studies have indicated that the activated LXR signaling pathway has an inhibitory effect on the proliferation of PDGF-BB-induced VSMCs.^447^ Notably, PDGF-BB stimulation was found to significantly upregulate ICAM-1 by VSMCs. Researchers prepared anti-ICAM-1 antibody-combined liposomes, for the delivery of a water-insoluble LXR agonist, and found that LXR agonist-liposomes inhibited VSMCs proliferation during atherogenesis by downregulating minichromosome maintenance complex component 6 (MCM6) expression and repressing phosphorylation of retinoblastoma.^136^ Additionally, docetaxel-LDEs treatment markedly downregulated anti-apoptotic Bcl-2, pro-apoptotic caspase 3, caspase 9, and Bax. In addition, the cell proliferation marker proliferating cell nuclear antigen (PCNA) was reduced by 40%.^403^ LDEs combined with paclitaxel significantly suppressed atherosclerotic plaques in rabbits with high-fat feeding.^448^ Besides, ginkgolide A (GA)-loaded AuNPs remarkably alleviated proliferation and migration of mouse VSMCs and sustained a long-term effect compared to AuNPs treatment alone. Furthermore, GA-AuNPs inhibited VSMC proliferation through alleviating the activation of ERK1/2 and downregulating the levels of superoxide anion.^330^

Proliferation and migration of VSMCs result in intimal hyperplasia that ultimately leads to vascular restenosis. Notably, antiproliferative agents targeting VSMCs have become promising therapies for preventing vascular restenosis,^449^ while nanoparticles have emerged as significant tools for sustained drug release. To date, several anti-proliferative drugs, such as rapamycin, paclitaxel, doxorubicin, honokiol, heparin, low molecular weight heparin (LMWH), curcumin, and 1α,25(OH)_2_D_3_ have been developed and applied for the treatment of vascular restenosis.^450,451^ However, their clinical application is seriously limited by poor solubility and side effects. To circumvent these problems, researchers have applied nanoparticles to deliver anti-proliferative drugs and achieved excellent results. For instance, rapamycin-loaded nanoparticles treatment securely and pronouncedly attenuated vascular stenosis in comparison to saline injection in a vascular restenosis porcine model.^452^ Notably, rapamycin was released rapidly within 3 days when dispersed in pluronic gel, while rapamycin-loaded PLGA NPs embedded in pluronic gel released rapamycin more slowly for over 4 weeks. Additionally, rapamycin-PLGA nanoparticles exhibited a longer anti-proliferative effect than free rapamycin in rat VSMCs and rat balloon injury models,^453^ while administration of rapamycin gel-like nanoparticles also alleviated apoptosis in VSMCs by inhibiting caspase-3/7 activity.^454^ Besides, rapamycin carried in polylactic acid, PLGA, or Eudragit RS nanoparticles significantly alleviated intimal hyperplasia in swine percutaneous transluminal coronary angioplasty (PTCA) models.^455^ Another study showed that paclitaxel or doxorubicin carried in paramagnetic nanoparticles targeted VSMCs and remarkably alleviated VSMCs proliferation in vitro.^350^ Paclitaxel-loaded polymeric nanoparticles achieved potentiation of anti-proliferative effect on rabbit VSMCs and reduced the neointimal area by 50% in balloon-injured rabbit iliac arteries compared to free paclitaxel.^456^ Wei et al. packaged honokiol in MSNs and assembled them into honokiol-MSNs, and found that they effectively inhibited VSMCs proliferation and migration through alleviating Smad3 phosphorylation.^441^ In another study, researchers employed layered double hydroxide (LDH) nanoparticles to deliver LMWH for the prevention of vascular restenosis, and found that LMWH-LDH nanoparticles were rapidly internalized by VSMCs and dramatically attenuated VSMCs proliferation and migration.^457^ Notably, the application of 17β-estradiol (17-βE), ω-3-polyunsaturated fatty acids (PUFAs), and C6-ceramide (CER) in the treatment of vascular restenosis is limited by their extensive protein binding and lipophilicity. Deshpande et al. developed a nanoemulsion rich in ω-3-PUFA which effectively delivers CER and 17-βE to VSMCs and ECs. Nanoemulsion containing 17-βE and CER inhibited ECs and VSMCs proliferation through regulating the MAPK signaling pathway and increasing pro-apoptotic caspase 3/7 activity, respectively. In addition, ω-3-PUFA significantly decreased growth factor-stimulated cellular proliferation,^458^ while 1α,25(OH)_2_D_3_-loaded PLGA nanoparticles inhibited inflammation or apoptosis-associated vascular stenosis by inhibiting the expression of IER-3, CD68, MCP-1, and HIF-1α.^459^ Another study showed that PLGA nanoparticles encapsulated α-elastin loaded with dexamethasone dipropionate extend drug release and potentiated elastase sensibility, thereby resulting in differentiation of VSMCs towards contractile phenotype.^460^

Additionally, retinoic acid (RA)-loaded nanoparticles were shown to effectively and safely promote angiogenesis and proliferation and alleviate apoptosis in ischemic stroke models.^461^ The carbon nanomaterial was generated by conjugating PEG with hydrophilic carbon clusters and covalently bonding deferoxamine (DEF-HCC-PEG). Treatment of intracerebral hemorrhage models with DEF-HCC-PEG reportedly improved their nuclear and mitochondrial genome integrity through protecting cells against both senescence and ferroptosis.^462^ On the other hand, thapsigargin-loaded nanoparticles protected HK-2 human kidney tubular epithelial cells against oxidative stress-induced cell death by activating Nrf2 and forkhead box O 1 (FOXO1).^463^

### Nanoparticle-mediated cell transplantation and EVs delivery

Among the various cell types, endothelial progenitor cells (EPCs), embryonic cardiomyocytes (eCMs), and embryonic stem cell-derived cardiomyocytes (ESC-CMs) have been identified as significant candidates for treating heart failure post-infarction. Notably, low retention of EPCs in the infarct area contributes to the poor curative effect of EPCs treatment. Nanoparticles are being developed for precise transplantation of stem cells, long-term tracking, and maintenance of therapeutic effects.

Researchers have used magnetic nanoparticles to enhance long-term engraftment of cells,^464^ whereas EPCs labeled with silica-coated IONs were found to dramatically suppress the infarction size and myocardial apoptosis under the guidance of an external magnet.^465^ Besides, eCMs and ESC-CMs-loaded SOMag5 magnetic nanoparticles generated 7- and 4.4-fold enhancement in cell engraftment rate at 2 and 8 weeks of treatment, respectively. In addition, grafted eCMs showed higher proliferation and lesser apoptosis under the guidance of 1.3 T magnet.^464^ Intriguingly, a previous meta-analysis highlighted the critical therapeutic role played by stem cell transplantation in stroke development, and revealed that SPIONs are critical tools for tracking stem cells migration.^466^ Previous studies have also shown that cell transplantation plays anti-inflammatory, anti-apoptosis, and angiogenesis roles in the prevention of ICH.^467–469^ On the other hand, embryonic stem cells (ESCs), neural precursors, and neural stem cells (NSCs) hold great potential for treating ICH. Human ESCs-derived spherical neural masses combined with IONs (IONs-ESCs-SNMs) dramatically improved ICH-induced brain injury by ameliorating the transportation of stem cells to the brain. Results from an in vivo study demonstrated that treatment of ICH rats with IONs-ESCs-SNMs mediated a significant downregulation of pro-inflammatory factors and alleviated accumulation of neutrophils and macrophages.^470^

The effects of nanoparticles, in combination with stem cell-derived EVs, have been extensively investigated in MI. Intriguingly, results from a previous study demonstrated that polymeric nanoparticles-mediated melatonin delivery potent the protective effect on adipose-derived mesenchymal stem cells (ADSCs) compared with melatonin alone.^325^ Moreover, melatonin-polymeric nanoparticles improved the survival rate of ADSCs and generated a more obvious therapeutic effect in the rat MI area compared to free melatonin. These results suggest that combining stem cell transplantation and melatonin-nanoparticles is a potential approach for MI treatment. Additionally, the introduction of magnetic nanoparticles has been associated with improved therapeutic efficiency of EVs, thus significantly reducing concerns related to low EVs production. For example, Lee et al. incorporated IONs with mesenchymal stem cells (MSCs) and prepared a novel exosome mimic extracellular nanovesicles (IONs-MSCs-EVs). The authors found that magnetic navigation induced IONs-MSCs-EVs localization to the infarcted heart and stimulated infarcted heart switch from inflammation phase to reparative phase, and also suppressed both fibrosis and apoptosis.^324^ In addition, magnetic nanoparticle composed of a Fe_3_O_4_ core and a PEG-coated silica shell collected circulating EVs via anti-CD63 and anti-myosin-light-chain on their surface. Under a local magnetic field and an acidic pH of an injured heart, the magnetic nanoparticles locally released EVs, thereby causing a reduction in the infarct area and improving angiogenesis and left-ventricle function.^471^ EVs secreted by pluripotent stem cells and their differentiated cardiomyocytes were also found to improve post-MI cardiac function. Additional evidence has indicated that injection of EVs markedly regulated hypoxic cardiomyocytes autophagy.^472^ On day 28 after MI, administration of cardiovascular progenitor cells-derived EVs promoted ECs migration and tube formation and ameliorated murine cardiac function.^473^

In transient middle cerebral artery occlusion (MCAO) mice models, engineered c(RGDyK) peptide-combined EVs were employed for curcumin delivery, where they strongly inhibited both inflammation and apoptosis.^474^ Neural progenitor cell-derived EVs showed intrinsic anti-inflammatory activity, whereas intravenous injection of RGD-combined EVs strongly inhibited inflammatory responses by suppressing the expression of the MAPK signaling pathway.^475^ In addition, Kim et al. demonstrated that IONs-MSCs-EVs significantly ameliorated ischemic-lesion targeting and the therapeutic outcome by promoting the production of therapeutic growth molecules. Notably, injection of IONs-MSCs-EVs and magnetic navigation mediated a 5.1-fold improvement in localization of nanomaterials to the ischemic lesion and further alleviated infarction size.^326^

Although glucocorticoids represent the main agents for kidney disease treatment, their clinical application is restricted by the occurrence of dose-dependent side effects, such as hyperglycemia and hypothalamic-pituitary-adrenal (HPA) axis suppression. A previous study showed that dexamethasone carried in macrophages-derived microvesicles (MVs) substantially suppressed renal injury by inhibiting renal inflammation and fibrosis, although low incidences of glucocorticoid-related side effects were observed after treatment.^476^ In another study, researchers used a MMP-2 sensitive self-assembling peptide (KMP2) hydrogel for the delivery of MSCs-derived EVs, and found that treatment with MSCs-EVs-KMP2 ameliorated renal function by downregulating the expression of pro-inflammatory cytokines, alleviating tubular cell apoptosis, and suppressing macrophage infiltration. Besides, MSCs-EVs-KMP2 administration was highly beneficial to proliferation and angiogenesis of ECs in mice with renal I/R injury.^477^

### Nanoparticle-mediated gene therapy

Epigenetic alterations are reversible. Therefore, prospecting for epigenome-affecting modalities represents an attractive research area to guide the development of interventions for the treatment of vascular aging-related diseases. Previous studies have described the role of small interfering RNAs (siRNAs) and short hairpin RNAs (shRNAs) in the management of disease progression via sequence-specific gene silencing.^478–480^ Notably, approximately 60% of human protein-coding gene expression is controlled by miRNAs. DNA fragments, siRNAs, miRNAs, and anti-miRNAs function as genetic drugs for the treatment of vascular aging-related diseases. However, their application is limited by enormous obstructions, such as rapid degradation in body fluids and potential off-target effects.^481^ Therefore, the development of effective drug delivery systems is imperative to efficient selective delivery to pathological tissues or cells. Currently available nucleic acid delivery systems are mainly classified into viral and non-viral categories.^482^ To date, however, their application has been limited by the potentially uncontrollable mutagenesis of virus-based vectors. Nanoparticles represent a novel type of non-viral carrier and a promising strategy that can be transfected in a sustained, targeted, and stable manner. Notably, nanoparticle-mediated delivery of gene drugs has been extensively investigated for the prevention and intervention of vascular aging-related disorders (Table 5).

**Table 5:** Nanoparticle-mediated gene therapies for vascular aging-related diseases

Diseases	Nanoparticles	Payload	Therapeutic effects	Ref(s)
Atherosclerosis	SNALPs	ApoB siRNA	Downregulate serum cholesterol, LDL, and ApoB protein levels	^485^
	Liposomes	ApoB siRNA	Decrease the expression of ApoB mRNA and protein, and serum LDL level	^494^
	Liposomes	ORC1 siRNA	Induce VSMCs enter to a reversible G(0) quiescent	^489^
	LNPs	PCSK9 siRNA	Reduce plasma cholesterol	^486^
	LNPs	CCR2 siRNA	Attenuate atherosclerosis by targeting inflammatory monocytes	^487^
	LNPs	LPA siRNA	Pronouncedly reduce the expression of LPA mRNA and lipoprotein(a)	^488^
	PLGA NPs	CaMKIIγ siRNA	Alleviate fibrous cap thickness and enhanced plaque stability by regulating the expression of CaMKIIγ and MerTK	^490^
	PLGA NPs	CCR2 shRNA	Effectively silence CCR2 gene in atherosclerotic macrophages	^493^
	HA NPs	LOX-1 siRNA	Decrease plaque area and lipid accumulation through inhibiting macrophage infiltration and MCP-1 expression	^483,484^
	p5RHH NPs	JNK2 siRNA	Rescue endothelial barrier integrity in atherosclerotic plaques by suppressing STAT3 and NF-κB	^491^
	mDNPs	SR-A siRNA	Significantly decrease the uptaken of ox-LDL	^492^
	Polymer-lipid hybrid NPs	Anti-miRNA-155	Inhibit atherosclerosis	^498^
	VHPK-CCLs	Anti-miRNA-712	Pronouncedly inhibited the activity of metalloproteinase	^495^
	Micelles	MiRNA-145	Enhance the expression of calponin, α-SMA, and myocardin	^42^
	PLGA NPs	MiRNA-145	Significantly inhibited VSMCs proliferation and prevented intimal hyperplasia	^43^
	Chitosan NPs	MiRNA-206, MiRNA-223	Enhance the expression of ABCA1 and reverse cholesterol transport	^496^
	GQDs	MiRNA-223	Regulate inflammation and attenuate plaque burden	^497^
Hypertension	LNPs	Angiotensinogen siRNA	Significantly and consistently reduced blood pressure	^503^
	PEG-PEI-Bu	Angiotensinogen shRNA	Inhibit hypertension by alleviating angiotensinogen expression	^504^
	Gal‑PEG-PEI	Angiotensinogen shRNA	Significantly inhibit hypertension through reducing the expression of angiotensinogen mRNA and protein, and the level of plasma angiotensinogen	^505^
Vascular restenosis	HB-OLD7	NOX2 siRNA	Inhibit neointima	^506^
	DA-PEI NPs	MMP-2 siRNA	Inhibit vascular restenosis	^508^
	PEG-Et 1:1	Smad3 shRNA	Inhibit intimal hyperplasia through suppressing the expression of collagen, MMP-1, MMP-2, and MMP-9 and enhancing the expression of TIMP1	^509^
	PLGA NPs	ICAM-1 siRNA	Accelerate ECs regeneration	^586^
	Chitosan NPs	PDGF-B siRNA	Inhibit the proliferation of VSMCs by reducing the expression of PCNA	^510^
	PEI NPs	Akt1 siRNA	Suppress VSMCs proliferation	^507^
	Magnetic nanospheres	VEGF plasmids	Inhibit intimal hyperplasia by enhancing the expression of exogenous VEGF	^587^
	PLGA NPs	VEGF gene	Remarkably decrease neointima area and cell proliferation	^511^
	PLGA NPs	VEGF plasmids	Promote reendothelialization and alleviate VSMCs proliferation	^512^
	PLGA NPs	Anti-MCP-1 gene	Significantly alleviate intimal hyperplasia	^588^
	Polymeric NPs	VEGF plasmids, ERK2 siRNA	Promote ECs proliferation/migration and attenuate VSMCs proliferation/migration	^513^
	PLGA NPs	MiRNA-145	Attenuate intimal hyperplasia through maintaining VSMCs in a contractile state	^43^
	Polymeric NPs	MiRNA-126	Pronouncedly potentiate HUVECs proliferation through downregulating the expression of SPRED1 and inhibit VSMCs proliferation by upregulating the level of IRS-1	^514^
MI	lipidoid NPs	CRMP2 siRNA	Improve infarct healing in experimental MI mice by reducing inflammation and fibrosis	^515^
	lipidoid NPs	IFR5 siRNA	Augment resolution of inflammation in healing infarcts by macrophage phenotype manipulation	^516^
	PMSNs	CCR2 siRNA	Improve the effectiveness of MSCs transplantation and selectively ameliorate myocardial remodeling after MI	^517^
	PAMAM NPs	PHD2 siRNA	Enhance the efficiency of stem cell transplantation for infarcted myocardium repair	^518^
	NPs	MiRNA-21	Possess protective effects on remote myocardium by alleviating inflammation, fibrosis, and cell apoptosis	^519^
	Gel@MSNs	MiRNA-21-5p	Effectively reduce infarct size through reducing inflammation and enhancing angiogenesis	^520^
	ADSC-exosome	MiRNA-31	Promote angiogenesis via miRNA-31/FIH1/HIF-1α pathway	^521^
	RGD-PEG-PLGA NPs	MiRNA-133	Inhibit cardiomyocyte apoptosis, inflammation, and oxidative stress by the regulation of the SIRT3/AMPK pathway	^522^
	Polymerics NPs	MiRNA-155-5p	Potentially increase an endogenous cytoprotective response and decrease damage within infarcted hearts	^523^
	Hep@PGEA NPs	MiRNA-499	Successfully explore for effective miRNA-pDNA staged gene therapy of MI	^525^
	Lipidoid NPs	ModRNA	Improve cardiac regeneration and function	^526^
	Chitosan-alginate NPs	PIGF	Enhance the positive effects of the growth factor in the setting of MI	^589^
Ischemic stoke	Alkyl-PEI/SPIO NPs	PHD2 siRNA	Improve EPC-based therapy efficacy for ischemic stroke	^527^
	FRS-NPs	ICAM-1 siRNA	Potentially applied for inhibition of inflammation in ischemic stroke	^528^
	CNTs	Caspase-3 siRNA	Recovery from brain ischemic insult	^529^
	PAMAM NPs	HGMB-1 siRNA	Exhibit neuroprotective effects on the postischemic brain	^530^
	NPs	MiRNA-195	Reduce the size of brain damage and improve functional recovery in stroke rats	^531^
Ischemic stoke	PLGA NPs	Anti-miRNA-141-3p	Significantly improve the effectiveness of anti-miRNA-141-3p	^532^
	Polymeric NPs	HO-1 plasmid	Significantly decrease cell death and infarct volume in the stroke models	^533^
	Dendrimer	HO-1 plasmid	Reduce apoptosis levels and infarct sizes in ischemic brains	^535^
	SPIONs	LncRNA Pnky siRNA	Enhance stem cell-based therapies for a stroke	^537^
	Polymeric NPs	HO1-mRNA, HO1-pDNA	Efficiently reduce infarct size	^534^
ICH	Tat-GS NPs	CGRP gene	Effectively attenuate vasospasm and improve neurological outcomes in an experimental rat model of subarachnoid hemorrhage	^538^
	PBCA NPs	Neurotrophin-3 plasmid	Inhibit the expression of apoptosis-inducing factor and reduce the cell death rate after ICH in vivo	^539^
CKD	PEI NPs	MiRNA-146a	Inhibit renal fibrosis in vivo	^540^

*SNALPs* stable nucleic acid lipid particles, *LDL* low-density lipoprotein, *VSMCs* vascular smooth muscle cells, *LNPs* lipid nanoparticles, *PLGA* poly lactic-co-glycolic acid, *HA* hyaluronic acid, *MCP-1* monocyte chemotactic protein, *STAT3* signal transducer and activator of transcription 3, *NF-κB* nuclear factor-kappaB, *mDNPs* mannose-functionalized dendrimeric nanoparticles, *ox-LDL* oxidized low-density lipoprotein, *CCLs* coated, cationic lipoparticles, *ABCA1* ATP binding cassette transporter A1, *GQDs* graphene quantum dots, *LNPs* lipid nanoparticles, *PEG* poly(ethylene glycol), *PEI* polyethylenimine, *HB-OLD7* amino-acid-based nanoparticle, *MMP-1* matrix metalloproteinase 1, *PEG-Et 1:1* polyethylene glycol-graft-polyethylenimine derivative, *TIMP-1* tissue inhibitor of metalloproteinase 1, *PLGA* poly lactic-co-glycolic acid, *PCNA* proliferating cell nuclear antigen, *VEGF* vascular endothelial growth factor, *ECs* endothelial cells, *HUVECs* human umbilical vascular endothelial cells, *MI* myocardial infarction, *PMSNs* photoluminescent mesoporous silicon nanoparticles, *MSCs* mesenchymal stem cells, *PAMAM* poly(amidoamine), *NPs* nanoparticles, *ADSCs* adipose-derived stem cells, *FIH1* Factor inhibiting HIF-1, *HIF-1α* hypoxia-inducible factor 1α, *SIRT3* sirtuin 3, *AMPK* adenosine monophosphate protein kinase, *PIGF* placental growth factor, *SPIONs* superparamagnetic iron oxide nanoparticles, *EPC* endothelial progenitor cell, *CNTs* carbon nanotubes, *HO1* heme oxygenase 1, *Tat-GS* Tat peptide-decorated gelatin-siloxane, *CGRP* calcitonin gene-related peptide, *PBCA* polybutylcyanoacrylate, *ICH* Intracerebral hemorrhage, *CKD* chronic kidney disease

#### Vascular aging-related cardiovascular diseases

Growing evidence suggested that nanoparticles encapsulated siRNAs, shRNAs, miRNAs, anti-miRNAs, and DNA fragments have effective, rapid, and durable therapeutic benefits for vascular aging-related cardiovascular diseases.^42,483,484^ The distribution of siRNAs such as ApoB siRNA, PCSK9 siRNA, LOX-1 siRNA, CCR2 siRNA, LPA siRNA, ORC1 siRNA, CaMKIIγ siRNA, p5RHH-JNK2 siRNA, SA-A siRNA, and CCR2 shRNAs via nanoparticles has been widely investigated in the prevention and intervention of atherosclerosis.^484–493^ For instance, intravenous administration of ApoB siRNAs-nanoparticles significantly downregulated serum cholesterol, LDL, and ApoB protein levels.^485,494^ These anti-atherosclerotic effects were observed 24-h after injection and sustained for 11 days at the highest dose.^485^ Additionally, nanoparticles-delivered miRNAs and anti-miRNAs, such as anti-miRNA-712, miRNA-206, miRNA-223, miRNA-155, miRNA-146a, miRNA-181b, and miRNA-145, are promising therapeutic approaches for atherosclerosis prevention.^42,43,495–500^ For instance, Chin et al. demonstrated in vitro that miRNA-145-combining micelles boosted the expression of atheroprotective contractile markers such as calponin, α-SMA, and myocardin. Moreover, miRNA-145 micelles alleviated 49% plaque growth and sustained an increased level of miRNA-145 after 2 weeks of treatment in the early atherosclerosis stage, whereas in the mid-atherosclerosis stage, miRNA-145 micelles ameliorated 43% and 35% lesion growth in comparison to free PBS and miRNA-145, respectively.^42^ Chitosan nanoparticle-encapsulated miRNA-33 specifically targeted macrophages and reduced ABCA1 expression, whereas chitosan nanoparticles cholesterol efflux-promoting miRNAs such as miRNA-206 and miRNA-223 increased ABCA1 expression and reversed cholesterol transport.^496^

The silencing of receptor genes that modulate blood pressure is referred to as gene therapy for hypertension. Numerous pieces of evidence suggest that siRNA-based therapeutic modalities are promising treatments for hypertension.^478,501^ Nanoparticles-based siRNA delivery systems can prevent siRNA from being degraded by endonuclease and exonuclease enzymes present in blood and cells.^502^ Olearczyk et al. developed a novel nanoformulation by conjugating angiotensinogen-specific siRNA with lipid nanoparticles. Angiotensinogen siRNA incorporated into lipid nanoparticles substantially decreased the levels of hepatic angiotensinogen mRNA of plasma angiotensinogen. In SHRs and Sprague-Dawley rats, intravenous injection of the conjugates significantly and consistently reduced blood pressure. Besides, the anti-hypertensive effect was maintained by weekly siRNA dosing.^503^ The PEG-PEI-Bu was employed in the delivery of angiotensinogen shRNA to normal rat liver cells to inhibit angiotensinogen expression in the treatment of hypertension.^504^ Additionally, biscarbamate‑crosslinked Gal‑PEG-PEI encapsulated angiotensinogen shRNA significantly inhibited hypertension by reducing angiotensinogen mRNA and protein expression, as well as plasma angiotensinogen levels.^505^

The siRNAs, such as NOX2 siRNA, Akt1 siRNA, MMP-2 siRNA, Smad3 shRNA, and PDGF-B siRNA have therapeutic effects in the treatment of vascular restenosis.^506–510^ After two weeks of treatment, the NOX2 siRNA-loaded amino-acid-based nanoparticle HB-OLD7 decreased NOX2 expression by over 87%. Furthermore, the neointima-to-media-area ratio and the lumen-to-whole-artery area ratio were reduced by over 83% and 89%, respectively.^506^ The MMP-2 siRNA functionalized with deoxycholic acid (DA) and encapsulated in PEI is an effective anti-restenotic treatment for atherosclerosis and vascular restenosis.^508^ In the rabbit iliac artery injury model, siRNA against PDGF-B loaded into chitosan nanoparticles significantly reduced the expression of PCNA and PDGF-B mRNA, reducing the proliferation of VSMCs.^510^ Additionally, vascular endothelial growth factor (VEGF) carried in nanoparticles significantly reduced neointima area and cell proliferation. The immunoreactivity of α-actin and PCNA were significantly lower after VEGF-nanoparticles administration.^511,512^ To create dual-targeting nanoparticles, grafted anionic polymers were surface functionalized with ECs-targeting REDV peptide and VSMCs-targeting VAPG peptide. The dual nanoparticles were used in the delivery of VEGF plasmids and ERK2 siRNA to promote ECs proliferation/migration and decrease VSMCs proliferation/migration, respectively.^513^ Besides, PLGA nanoparticles encapsulating miRNA-126-double strand RNA (dsRNA) pronouncedly potentiated human umbilical vascular endothelial cells (HUVECs) proliferation and migration by downregulating SPRED1 expression and attenuating VSMCs proliferation and migration through upregulating the IRS-1 levels.^514^

Nanoparticle-mediated gene therapies have also shown considerable potential for treating MI and heart failure. The administration of CRMP2 siRNA or IFR5 siRNA to infarcted hearts induced the M1 macrophage phenotype to switch to M2, remarkably reducing the inflammation and fibrosis in post-MI mice.^515,516^ The CCR2 siRNA carried by photoluminescent MSNs (PMSNs) reduced the inflammatory monocyte accumulation in infarcted lesions. Intriguingly, nanoparticles encapsulated with either CCR2 siRNA or PHD2 siRNA could be applied for enhancing the therapeutic efficiency of post-MI MSCs transplantation.^517,518^ Additionally, nanoparticles have been successfully transferred to a wide range of miRNAs in the infarcted heart, including miRNA-21, miRNA-21-5p, miRNA-31, miRNA-133, miRNA-155-5p, miRNA-199a-3p, and miRNA-499.^519–525^ Intravenous administration of miRNA-21 mimic-loaded nanoparticles induced cardiac macrophages to switch from pro-inflammatory phenotype to reparative phenotype and facilitated angiogenesis, and attenuated myocardium hypertrophy, fibrosis, and apoptosis.^519^ The MSNs were able to transfer miRNA-21-5p to the infarcted heart, which suppressed M1 macrophage polarization and promoted angiogenesis.^520^ Turnbull et al. reported that lipidoid nanoparticles containing modRNA enhanced cardiac regeneration and function in pig and rat myocardium.^526^

#### Vascular aging-related cerebrovascular diseases

Gene therapies in the treatment of ischemic stroke have attracted a lot of attention. It has been shown that siRNAs encapsulated in nanoparticles, such as PHD2 siRNA, ICAM-1 siRNA, caspase-3 siRNA, and HGMB-1 siRNA have effective therapeutic effects on ischemic stroke.^527–530^ Wang et al. found that nanoparticle-mediated PHD2 siRNA administration promoted EPCs survival and migration by increasing HIF-1α and C-X-C chemokine receptor type 4 (CXCR4) expressions. It provided an effective strategy for improving EPCs-based cell transplantation therapy for ischemic stroke.^527^ Furthermore, nanoparticle-mediated miRNAs administration, including miRNA-195, and anti-miRNA-141-3p, was successful in regulating ischemic stroke.^531,532^ MiRNA-195 carried in nanoparticles exhibited excellent anti-apoptotic, anti-inflammatory, pro-proliferation, and pro-migration capabilities in stroke mice models. The delivery of miRNA-195 improved brain function damage.^531^ Besides, there are particularly strong data that the HO-1 plasmid has a great anti-apoptotic and anti-inflammatory effect on stroke.^533,534^ Given its low cytotoxicity and high gene delivery efficiency, polyamidoamine generation 2 dendrimer was employed for HO-1 plasmid delivery and attenuated apoptosis in MCAO-reperfusion stroke animal models.^535,536^ Additionally, Lin et al. prepared a theranostic nanomedicine by combining SPIONs with siRNA-against Pnky lncRNA. This nanoformulation induced neuronal differentiation of neural stem cells and facilitated tracking of neural stem cells through Pnky lncRNA silencing and MRI, respectively.^537^

Notably, nanoparticles provide a promising approach for the safe and effective delivery of therapeutic genes in ICH. in a subarachnoid hemorrhage rat model, the Calcitonin Gene-Related Peptide (CGRP) gene carried in Tat peptide-modified gelatin-siloxane (CGRP-Tat-GS) significantly alleviated cerebral vasospasm and improved neurological function compared to single CGRP gene administration.^538^ Furthermore, after neurotrophin-3 plasmid-polybutylcyanoacrylate (PBCA) nanoparticles, ICH rats had increased expressions of neurotrophin-3 and reduced production of apoptosis-inducing factors.^539^

#### Vascular aging-related chronic kidney disease

Renal fibrosis is an end-stage renal disorder. Renal fibrosis suppression is crucial in improving the prognosis of patients with chronic kidney disease. However, no treatment for renal fibrosis has been established. Therefore, effective approaches for the intervention and prevention of renal fibrosis are necessary. Nanoparticle-mediated gene therapies provide a broad prospect for renal fibrosis treatment. The PEI nanoparticles containing miRNA-146a significantly suppressed TNF-β1/Smad and tumor necrosis factor receptor-associated factor 6 (TRAF-6)/NF-κB signaling pathways. Moreover, miRNA-146a administration suppressed α-smooth muscle actin expression, macrophage infiltration, and renal fibrosis area.^540^

## Clinical trials of nanoparticle

In view of the tremendous benefits and potential of nanoparticles, diagnostic and therapeutic methods based on nanoparticles have been developed for clinical use as contrast agents or drug vehicles. Many clinical trials have demonstrated the improved therapeutic efficacy of nanoparticles in vascular aging-related diseases (Table 6). However, many of them are still in their initial phases. Kharlamov et al. examined 180 patients with CAD in a multi-center, open-label, observational, and three arms study and revealed that plasmonic photothermal therapy using silica-AuNPs correlated to significant regression of coronary atherosclerosis.^541^ The total atheroma volume decreased by an average of 60.3 mm in the group with 12 months silica-AuNPs treatment. Compared to other groups, the risk of cardiovascular death in the nanoparticles group was much lower. Another randomized controlled trial demonstrated that prednisolone carried in liposomes had a better pharmacokinetic profile in humans, with the plasma half-life being enhanced to 63 hours.^542^ In a human trial involving 14 patients with acute ST-elevation MI (STEMI), USPIO-based contrast agents showed the potential for more precise characterization of infarcted lesions by identifying macrophage infiltration. In addition, USPIO-based contrast agents are more versatile and safer than gadolinium-based compounds.^292^ As per the observations from the PROTECT-TIMI 30 Trial (NCT00250471), using the nanoparticle cTnI test to identify myocardial injury in patients with unstable angina provides above 10 folds increase in analytical sensitivity compared with traditional generation cTnI tests.^543^ The incidence of major adverse cardiac events was lower in the prostaglandin E1-encapsulated liposomes treatment group than in the control group among 68 STEMI patients.^544^ Within 24-h of symptom onset, multimodal stroke imaging was performed in 12 patients with typical clinical symptoms, and USPIO-dependent signal changes were found to be spatially heterogeneous, reflecting different patterns of macrophage infiltration in different types of lesions. In stroke patients, USPIO-enhanced MRI may offer a specific target for anti-inflammatory treatments.^290^ These compelling observations open up new avenues for the clinical application of nanoparticles.

**Table 6:** Nanoparticles in clinical trial for diagnosis or therapy of vascular aging-related diseases

Diseases	Nanoparticles	Cargos	Outcome/purpose	ClinicalTrials.gov Identifier	Phase
Atherosclerosis	Silica-AuNPs	None	Reduce the total atheroma volume follow 12 months treatment	NCT01270139	Completed
	^64^Cu-25%-CANF-Comb	None	Demonstrate feasibility of PET imaging of radiopharmaceutical nanoparticle ^64^Cu-25%CANF-Comb uptake by PET-MR	NCT02417688	Phase 2
	LDE	Methotrexate	Evaluate the safety and efficacy of methotrexate-LDE in patients with stable coronary disease	NCT04616872	Phase 2
	LDE	Paclitaxel	Evaluate the safety and efficacy of paclitaxel-LDE in patients with stable coronary disease	NCT04148833	Phase 3
	PEG-liposome	Prednisolone	Continuous low-dosed anti-inflammatory drugs have great potential as novel treatment strategies	NCT01601106	Phase 2
MI	USPIOs	None	Hold major promise as a potential method for assessing cellular myocardial inflammation and left ventricular remodeling	NCT01323296	Completed
	USPIOs	None	Examine the ability of USPIOs to image myocardial inflammation following acute MI	NCT01995799	Phase 2
	Polymeric nanoparticles	BP-SES, DP-EES	Comparable safety and efficacy profiles of BP-SES and DP-EES were maintained throughout 2 years of follow-up	NCT01443104	Completed
	Ultra-sensitive nanoparticle	None	Improved analytical performance at very low concentrations of troponin	NCT00250471	Phase 3
	Nanoemulsion	Methotrexate	Evaluate the effect of methotrexate carried in nanoemulsion on left ventricular remodeling after STEMI	NCT03516903	Phase 2
	GQDs	None	Evaluate the sensitivity, precision, and effectiveness of photoelectrochemical immunosensor for early diagnosis of acute MI	NCT04390490	NA
Coronary artery disease	SPIONs	None	Evaluate the accuracy and safety of coronary artery contrast-enhanced MRI With polysaccharide SPIONs	NCT05032937	Phase 1
	Liposome	Alprostadil	Observe the safety and tolerability of single/multiple-dose administration of different doses of Alprostadil Liposome for Injection as well as to confirm the safety dose range	NCT02889822	Phase 1
Vascular restenosis	Albumin-nanoparticles	Paclitaxel	Determine the appropriate dose of the new medicine for future trials	NCT 00093223	Completed
	Albumin-nanoparticles	Paclitaxel	Investigate the use of systemic intracoronary administration of albumin-bound paclitaxel, ABI-007, for the prevention and reduction of restenosis	NCT00124943	Phase 2
	liposomes	Alendronate	Reduce in-stent restenosis as compared to placebo	NCT00739466	Completed
Chronic kidney disease	SPIONs	None	Explore the effectiveness and safety of polysaccharide SPIONs injection for contrast-enhanced renal artery magnetic resonance	NCT05045872	Phase 1
	liposomes	Iron	Evaluate the efficacy of treatment with liposomal oral iron compared to intravenous iron in chronic kidney disease anemic patients	NCT01864161	Phase 4

*AuNPs* gold nanoparticles, *PET* positron emission tomography, *MR* magnetic resonance, *LDE* cholesterol-rich non-protein nanoparticle, *MI* myocardial infarction, *USPIOs* ultrasmall superparamagnetic iron oxide nanoparticles, *BP-SES* biodegradable polymer sirolimus-eluting stents, *DP-EES* durable-polymer everolimus-eluting stents, *STEMI* ST-elevation myocardial infarction, *GQDs* Graphene quantum dots, *NA* not available, *SPIONs* superparamagnetic iron oxide nanoparticles, *MRI* magnetic resonance imaging

## Conclusion and future perspectives

This study aims to investigate and improve the understanding of the functions of various nanoparticle-based strategies in the diagnosis and treatment of vascular aging-related diseases, as well as spark some new ideas for researchers who are interested in nanoparticle-based clinical diagnosis and therapy techniques in multiple vascular disorders, even in other fields. Nanoparticles play crucial roles in the diagnosis and treatment of vascular diseases due to their unique optical and electrochemical properties. Herein, we discuss the classifications of nanoparticles and the mechanisms of vascular aging. Importantly, we have extensively reviewed nanoparticle-based strategies in vascular aging-related diseases. As a diagnostic tool, nanoparticles have the potential to improve diagnostic efficiency and accuracy. On the one hand, nanoparticles as biosensors can detect specific biomarkers in plasma, serum, and urine in a sensitive and stable manner. On the other hand, nanoparticles as contrast agents can be designed and manipulated to visualize typical pathological changes in diseases such as inflammation, thrombosis, angiogenesis, proliferation, and apoptosis. In terms of clinical therapy, nanoparticles as antioxidant and anti-proliferative agents, as well as drug delivery vesicles are being studied extensively for the treatment of vascular aging-related diseases such as cardiovascular diseases (e.g., atherosclerosis, hypertension, vascular restenosis, MI, and heart failure), cerebrovascular diseases (e.g., ischemic stroke, ICH, and vascular dementia), and chronic kidney disease.

**Fig. 7:**
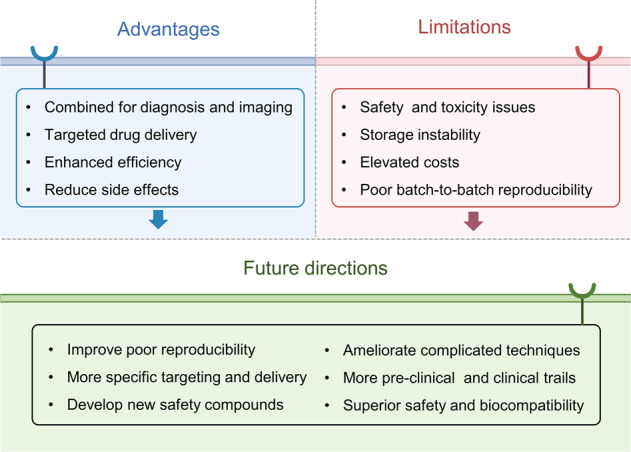
Advantages, limitations, and future directions of nanomedicine

The advancement of nanoparticle diagnostic and therapeutic applications has potentially transformed the diagnosis and treatment paradigm of vascular aging and related diseases (Fig. 7). However, development in the applications of nanoparticles in vascular diseases is predominantly limited to basic research. Over the past two decades, numerous nanomedicines have been approved by FDA or have shown promise for future clinical transformation. In this case, the safety and toxicity issues of nanoparticles are critical concerns in clinical use.^545^ Notably, several approved nanomedicines such as Doxil and Abraxane show fewer side effects than their small-molecule counterparts, while magnetic and carbon-based nanoparticles tend to display toxicity.^546–549^ Many mesotheliomas and lung cancers have been linked to asbestos exposure, raising concerns about the potential carcinogenicity of high aspect ratio nanoparticles such as CNTs.^550^ It has been reported that silica nanoparticle exposure is associated with adverse cardiovascular effects. For example, Wang et al. demonstrated that silica nanoparticles induced pyroptosis and cardiac hypertrophy via the ROS/NLRP3/Caspase-1 signaling pathway.^551^ The AuNPs have been extensively studied in the biomedical field, however, AuNPs with diameters less than 2 nm exhibit cytotoxic profile.^552^ Moreover, the size of nanoparticles affected their distribution, ultrasmall AuNPs have significantly longer circulation duration and distinct biodistributions in comparison to larger AuNPs.^553^ Enea et al. examined the cytotoxicity induced by AuNPs with various shapes (nanostars and nanospheres) and sizes (15 nm and 60 nm). Despite the low toxicity of AuNPs, the smaller 15 nm AuNPs spheres sized have the highest toxicity among all tested AuNPs.^554^ The toxicity of nanoparticles is directly related to the depletion of the intracellular antioxidant pool, the generation of endogenous ROS, oxidative stress, and the disruption of immunological responses and cellular components.^555^ Additionally, diverse administration routes also show varying toxicity. According to research, the oral and inhalation routes have higher toxicity than injection. Indeed, organ systems that include the nervous system, thyroid, heart, lungs, mononuclear phagocytic system, and even the reproductive system exhibited potential toxic effects after being injected with IONs-formulations.^547^ Assessing the toxicity of nanoparticles remains a challenge, especially in vivo evaluation and long-term toxicity studies.^556^ Another barrier to clinical applications of nanoparticles is their sophisticated constructions, which include diverse surface modifications and multiple payloads, resulting in elaborate manufacturing and quality control processes, storage instability, enhanced costs, and poor batch-to-batch reproducibility. All of these problems impede large-scale production. Furthermore, the sector of nanomedicines entering the market is progressing at a snail's pace due to the long period it takes to conduct preclinical and clinical studies.^557^ Despite all the positive outcomes achieved in cell and animal model studies, several limitations need to be solved before nanoparticles can be used in clinical applications. Further research on biosensors for diagnosis should focus on improving the poor reproducibility and complicated procedures.^8^ Similarly, future research on nanoparticle-based drug administration should include more specific targeting and delivery, superior safety and biocompatibility, reduced toxicity while maintaining therapeutic efficacy, and the development of novel safety compounds. Special attention should be given to experimenting on animals with diseases representing human socially serious illnesses. Researchers should strive to elucidate the mechanisms of action, biodistribution, and bioaccumulation, as well as possible short-term and long-term adverse effects of these nanoparticles.

Therefore, substantial investigations remain to be completed before nanoparticles can be used in the clinical diagnosis and treatment of vascular aging-related diseases. Despite the existing limitations, a lot of research suggests that nanoparticles have the potential for treating vascular aging-related diseases. Comprehensive knowledge of the pathogenesis of vascular aging may lead to the identification of new biomarkers and therapeutic targets, providing new insights toward future vascular aging treatment. The advancement in nanotechnology has resulted in an amazing revolution in the diagnosis and treatment of vascular aging-related diseases.
